# Design, synthesis, antiviral activities of ferulic acid derivatives

**DOI:** 10.3389/fphar.2023.1133655

**Published:** 2023-03-03

**Authors:** Jin-long Mao, Lei Wang, Shu-jie Chen, Bin Yan, Li-ying Xun, Rui-cheng Li, Pei-chen Wang, Qi-tao Zhao

**Affiliations:** ^1^ School of Pharmacy, Shandong University of Traditional Chinese Medicine, Jinan, Shandong, China; ^2^ School of Traditional Chinese Medicine, Shandong University of Traditional Chinese Medicine, Jinan, Shandong, China

**Keywords:** ferulic acid, diphenyl acrylic acids, *cis-trans* isomers, antiviral, RSV

## Abstract

A series of novel ferulic acid derivatives were designed and synthesized, and the twenty-one compounds were evaluated for their antiviral activities against Respiratory syncytial virus (RSV), herpes simplex virus type 1 (HSV-1), and enterovirus type 71 (EV71). These derivatives with the core structure of diphenyl acrylic acids had *cis-trans* isomers, which were confirmed by ^1^H NMR, HPLC, and UV-vis spectra for the first time. The A5 had a selective effect against RSV but no work on herpes simplex virus type 1 and enterovirus type 71, which showed a therapeutic index (TI) of 32 and was significantly better than ferulic acid. The A5 had no scavenging effect on free radicals, but the A2 as the degradation of A5 showed an obvious scavenging effect on DPPH· and ABTS^+^·. In addition, the A5 had no toxicity to endothelial cells and even showed a proliferative effect. Therefore, the A5 is worth further optimizing its structure as a lead compound and investigating the mechanism of inhibiting Respiratory syncytial virus.

## 1 Introduction

Respiratory syncytial virus (RSV) can cause severe lower respiratory tract infections and is one of the major reasons for illness and even death in the young children, elderly and immunocompromised adult (Nam and Ison, 2019; Falsey et al., 2022). There is still no safe and effective RSV vaccine approved for use, only palivizumab is available to prevent RSV infection in high-risk infants (Li et al., 2021). Ribavirin, a broad-spectrum antiviral drug, is approved for the treatment of RSV infection, but has shortcomings such as long treatment cycles and large adverse effects, especially in patients with significant cardiac symptoms, which can easily lead to cardiovascular complications (Ivey et al., 2018). Oxidative stress is one of the pathological bases of cardiovascular diseases, and it is beneficial to improve the safety of anti-RSV drugs if the vascular endothelial function is enhanced by increasing the antioxidant activity of these drugs. Currently, there are a few drugs available for the clinical treatment of RSV infection, and the use of natural products as lead compounds is one of the effective tactics to discover new antiviral drugs.

Ferulic acid (FA) is one of the active components of traditional Chinese medicine, such as *Ferula assafoetida* L. and *Angelica sinensis* (Zhang and Gao, 2020; Al-Mutairi et al., 2021; Wang et al., 2021). FA has a variety of biological activities, such as antioxidant (Bian et al., 2015; Lambruschini et al., 2021), anti-inflammatory (Montaser et al., 2019; Yin et al., 2019), antibacterial (Ibitoye and Ajiboye, 2019), anticancer (Gao et al., 2018), antiviral (Antonopoulou et al., 2022), and cardiovascular protective effects (Zhou et al., 2017). Liu et al. (2015) designed and synthesized non-nucleoside acyl oximes of FA with good antiviral activity against the hepatitis B virus (HBV), which inhibited the secretion of HBsAg and HBeAg in HepG 2.2.15 cells, and inhibited the replication of HBV DNA. Wang et al. (2017) synthesized the ferulic acid acylhydrazone derivatives which showed good antiviral activity against the tobacco mosaic virus (TMV), and had good binding ability to amino acid residues on the TMV coat protein (TMV-CP). Cui et al. (2019) synthesized the amide derivatives of FA that inhibited the neuraminidase (NA) activity of influenza virus H1N1, and the 4-OH-3-OMe and the amide (CON) groups were the key pharmacodynamic groups for the inhibition of NA activity. Sakai et al. (1999) found that inflammatory protein-2 (MIR-2) was produced after RSV infected the macrophage RAW264.7, and the presence of FA decreased MIP-2 levels, thereby reducing neutrophil infiltration into the site of inflammation, which suggested that FA had a therapeutic effect on inflammation induced by RSV induction.

FA is a natural product with antiviral activity (Antonopoulou et al., 2022). In this experiment, novel ferulic acid derivatives with the core structure of diphenyl acrylic acids were synthesized to get 21 compounds and confirmed by MS, IR, ^1^H NMR or ^13^C NMR, most of these structures were not reported in the Reaxys database. There are *cis-trans* isomers in these derivatives, and the conformational analysis was firstly performed by ^1^H NMR, HPLC and UV-vis spectra. These derivatives were evaluated for their antiviral activities against respiratory syncytial virus (RSV), herpes simplex virus type 1 (HSV-1) and enterovirus type 71 (EV71), the A5 had a selective effect against RSV, with better activity than ribavirin and FA. These derivatives were also investigated for their antioxidant activities and vascular endothelial protective effects.

## 2 Results and discussion

### 2.1 Chemical

#### 2.1.1 Design and synthesis of target compounds

**FIGURE 1:**
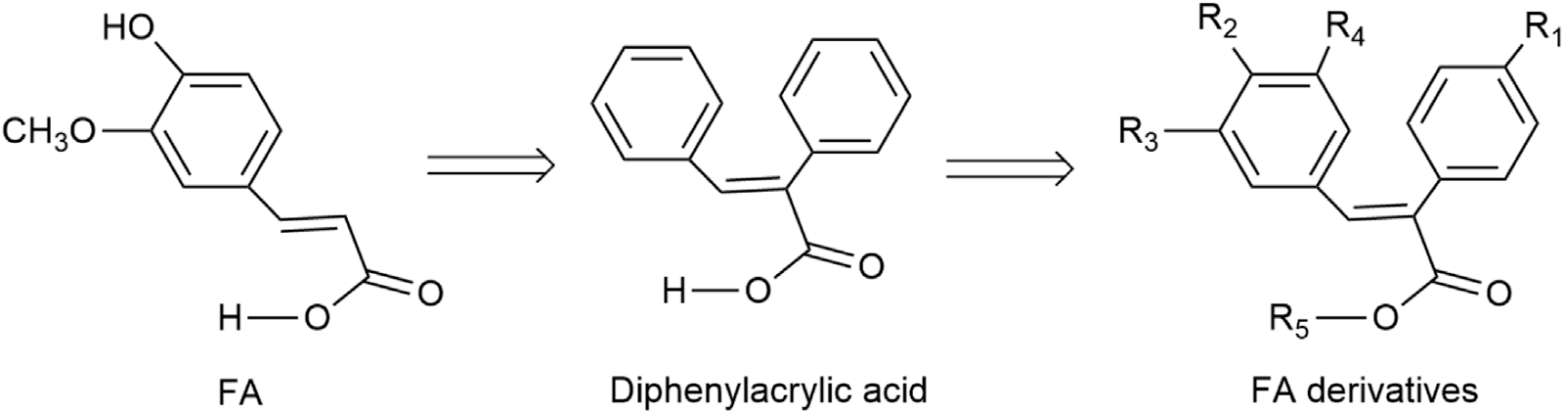
The designed structure of FA derivatives.

These novel FA derivatives are outlined in Figure 1 and Table 1. The designed target is 2,3-diphenyl acrylic acids, namely, two aryl groups are attached to the ethylene bridge, so there are *cis-trans* isomers. These derivatives were synthesized using the Perkin reaction between benzaldehydes and phenylacetic acids, which was known to favor of the (E)-isomer. Many studies only reported that (E)-isomers were isolated (Moreau et al., 2006; Burja et al., 2010; Gazvoda et al., 2013), and we also mainly obtained (E)-isomers when the pH of the solution was about 6–7. In addition, a small amount of (Z)-isomers were unexpectedly isolated when the pH was adjusted to about 2–3. The *cis-trans* isomers in these cases were compared by ^1^H NMR, HPLC and UV-vis spectra for the first time.

**TABLE 1:** Structures of FA derivatives and their inhibitory effects on RSV, HSV-1, and EV71 viruses.

Ferulic acid derivatives							Antiviral effect		
structure		R1	R2	R3	R4	R5	RSV	HSV-1	EV71
Ribavirin							16	16	8
FA							1	1	1
A1	(*E*)-	-SO_2_CH_3_	-OH	-OCH_3_	-H	-H	4	1	1
A2	(*E*)-	-SO_2_CH_3_	-OH	-OCH_3_	-H	-CH_2_CH_3_	16	1	1
A3	(*E*)-	-SO_2_CH_3_	-OCH_3_	-OCH_3_	-H	-H	1	1	1
A4	(*E*)-	-SO_2_CH_3_	-OCOCH_3_	-OCH_3_	-H	-H	1	1	1
A5	(*E*)-	-SO_2_CH_3_	-OCOCH_3_	-OCH_3_	-H	-CH_2_CH_3_	32	1	1
A6	(*Z*)-	-SO_2_CH_3_	-OH	-OCH_3_	-H	-H	8	1	1
A7	(*E*)-	-H	-OH	-OCH_3_	-H	-H	8	1	2
A8	(*E*)-	-H	-OCH_3_	-OCH_3_	-H	-H	8	1	2
A9	(*E*)-	-H	-OCOCH_3_	-OCH_3_	-H	-H	2	1	1
B1	(*E*)-	-SO_2_CH_3_	-OH	-H	-H	-H	4	8	1
B2	(*E*)-	-SO_2_CH_3_	-OH	-H	-H	-CH_2_CH_3_	4	1	1
B3	(*E*)-	-SO_2_CH_3_	-OCOCH_3_	-H	-H	-CH_2_CH_3_	2	1	1
B4	(*Z*)-	-SO_2_CH_3_	-OH	-H	-H	-H	8	1	1
C1	(*E*)-	-SO_2_CH_3_	-OH	-Br	-H	-H	1	1	1
C2	(*Z*)-	-SO_2_CH_3_	-OH	-Br	-H	-H	1	1	2
C3	(*E*)-	-SO_2_CH_3_	-OCOCH_3_	-Br	-H	-H	2	8	2
C4	(*E*)-	-SO_2_CH_3_	-OH	-OCH_3_	-Br	-H	1	1	1
C5	(*E*)-	-SO_2_CH_3_	-OCOCH_3_	-OCH_3_	-Br	-H	2	8	2
C6	(*E*)-	-SO_2_CH_3_	-OH	-OCH_3_	-H	-Na	2	1	2
C7	(*E*)-	-SO_2_CH_3_	-OCOCH_3_	-OCH_3_	-H	-Na	2	1	1
C8	(*E*)-	-SO_2_CH_3_	-ONa	-OCH_3_	-H	-CH_2_CH_3_	2	1	2

**SCHEME 1:**
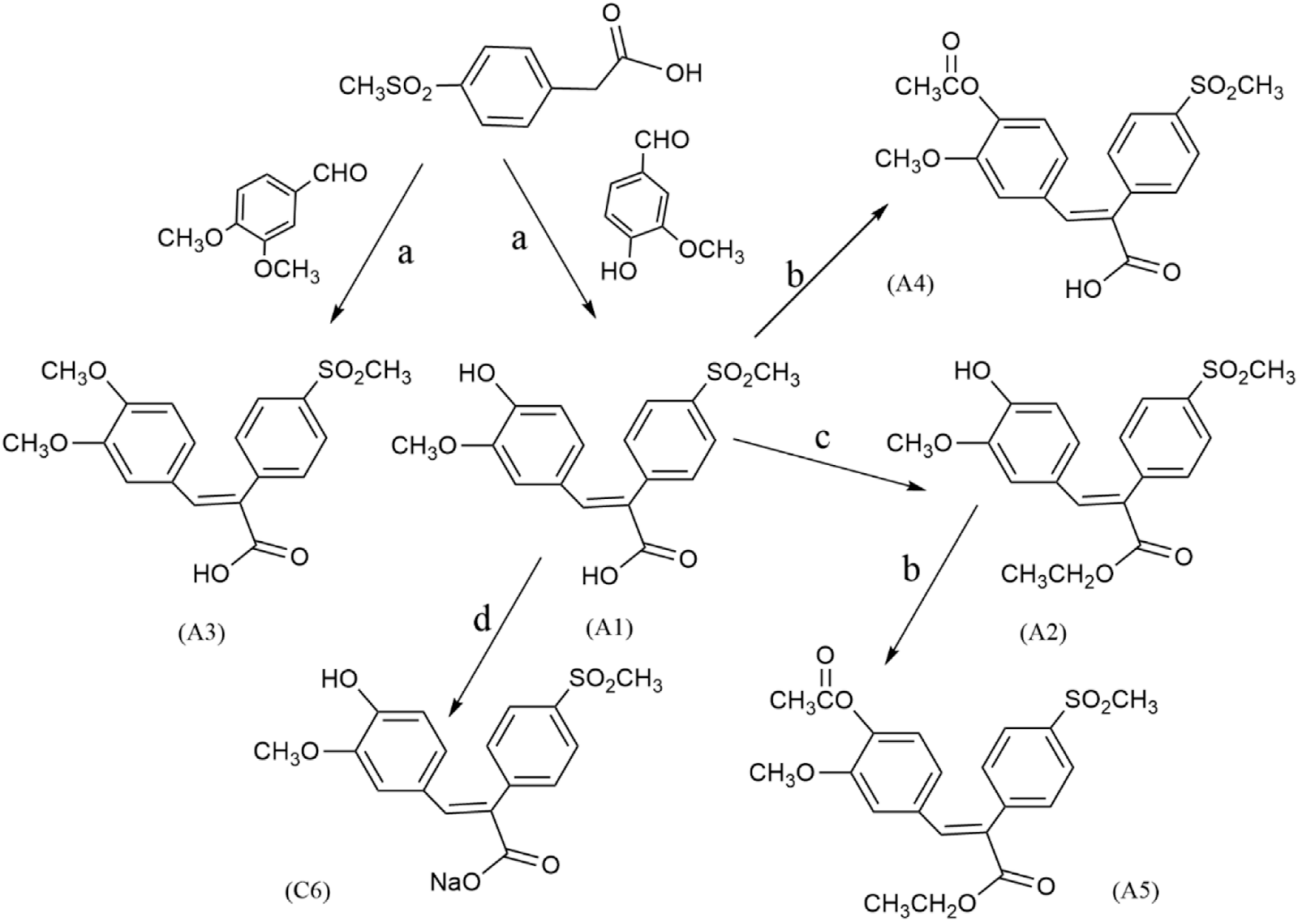
The general reaction route of FA derivatives. Reagents and conditions: **(a)** Et_3_N, Ac_2_O, 130°C, 3–5 h; **(b)** Ac_2_O, reflux, 3 h; **(c)** EtOH/H_2_SO_4_, reflux, 6 h; **(d)** NaHCO_3_, lyophilize.

The general route for preparing FA derivatives was shown in Scheme 1. We started with Perkin condensation of benzaldehydes and benzaldehydes in the presence of acetic anhydride and triethylamine under refluxing for about 3–5 h to give the 2,3-diarylpropenoic acids as final products. The solid residues were put into water for hydrolysis, after cooling and precipitation, recrystallizing from acetic acid, which mainly afforded the (E)-isomers such as A1, A3, B1, C1, and C4. The A1 and B1 reacted under reflux with anhydrous ethanol in the presence of concentrated sulfuric acid to give the esterified products A2 and B2 respectively. The A1, A2, B2, C2, and C4 were acylated with acetic anhydride under reflux to give the acylated products A4, A5, B3, C3, and C5, respectively, wherein A5 and B3 were simultaneously acylated and esterified. The A1 and A4 reacted with an equimolar NaHCO_3_ aqueous solution, respectively, and were lyophilized to give the carboxylate salts C6 and C7. The A2 was dissolved in an equimolar NaOH aqueous solution under heating, and then lyophilized to give the phenoxide salt C8. When the solution after precipitating the (E)-isomer such as A1, B1 or C1 was acidified with diluted HCl, the related (Z)-isomer such as A6, B4 or C2 was isolated in small amounts at a pH of about 2–3.

#### 2.1.2 Structural studies of FA derivatives

**FIGURE 2:**
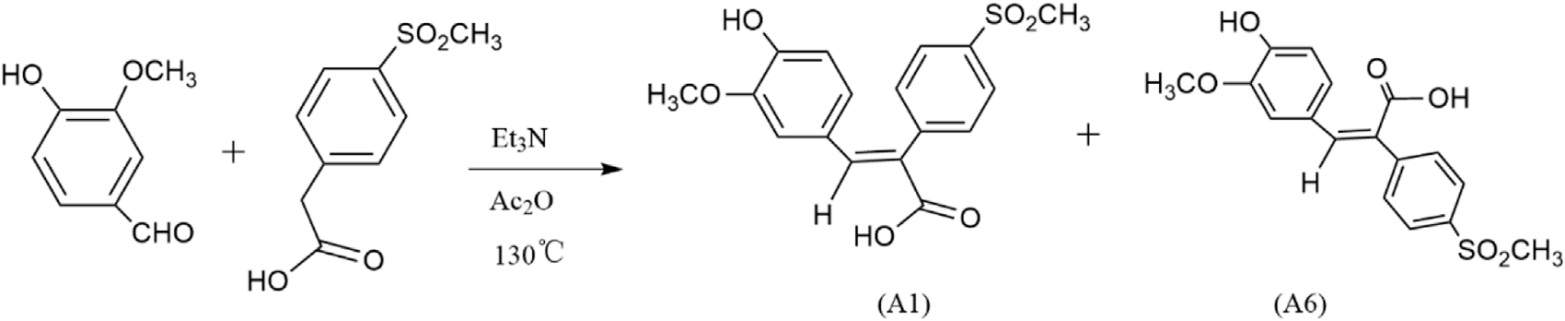
Synthesis of *cis-trans* isomers A1 and A6. (After reacting 4 h at Et_3_N, Ac_2_O, 130 °C, a small amount of water was added, and then dilute NaOH was added. Dilute HCl adjusted the pH at 6-7 to obtain A1, and adjusted the pH at about 2-3 to obtain A6.)

**FIGURE 3:**
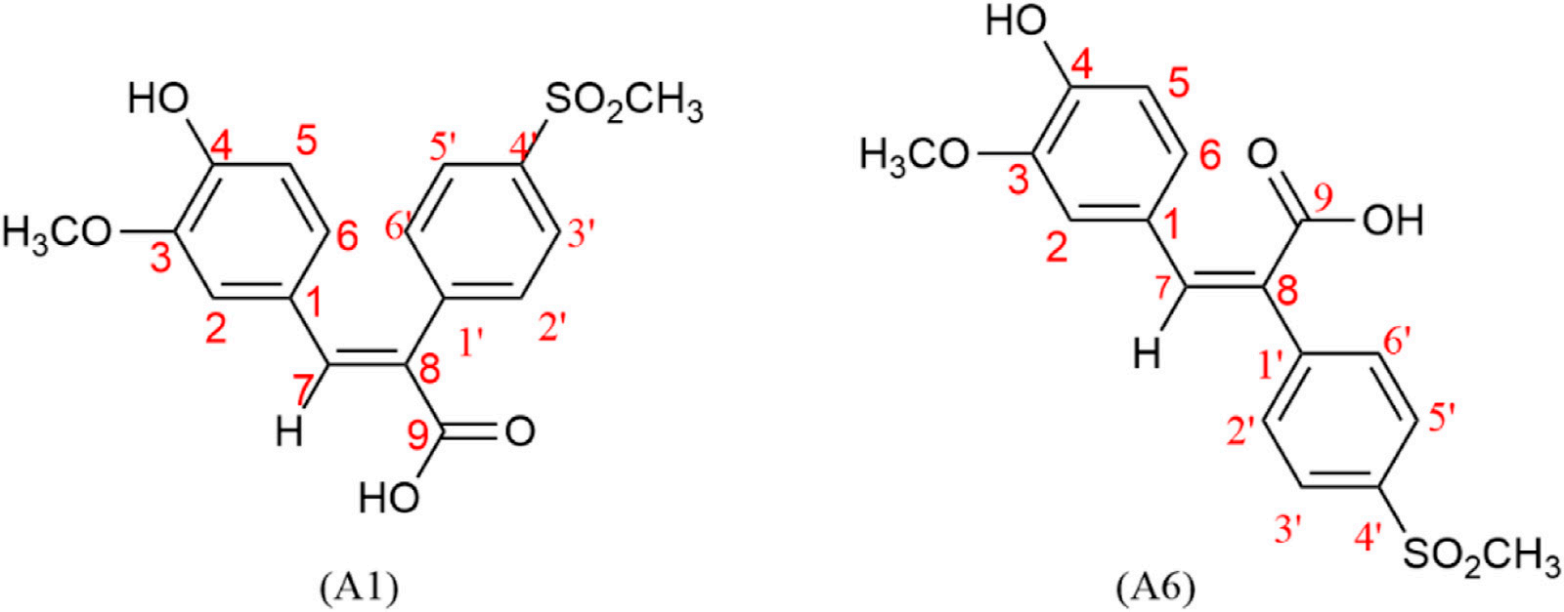
Numbering of hydrogen on *cis-trans* isomers A1 and A6.

A series of novel FA derivatives with the core structure of diphenyl acrylic acids were synthesized and their structures were confirmed by MS, IR, ^1^H NMR or ^13^C NMR, *etc*. Some diphenyl acrylic acids have been mentioned in the literature, such as A2, A4 (Mao et al., 2011; Mao et al., 2014), B1 (Moreau et al., 2006; Gazvoda et al., 2013), A7, A8, A9 (de Lima et al., 2009; Ullah et al., 2019), but the synthesis or characterization details were partially available. These derivatives exist in *cis-* or *trans-*isomer, and the determination of the *cis-trans* isomerism is one of the keys to structural studies. In the previous studies, the A1 has been synthesized and confirmed to be the *cis*-isomer or (E)-isomer by HMQC and NOESY (Mao et al., 2011). Now, the A6 as the *trans*-isomer or (Z)-isomer of A1 was isolated and confirmed (Figures 2, 3), and the *cis-trans* isomerism of A1 and A6 was further compared by ^1^H NMR, HPLC and UV-vis spectra.

##### 2.1.2.1 The double-bonded hydrogen of *cis-trans* isomers in ^1^H NMR

**FIGURE 4:**
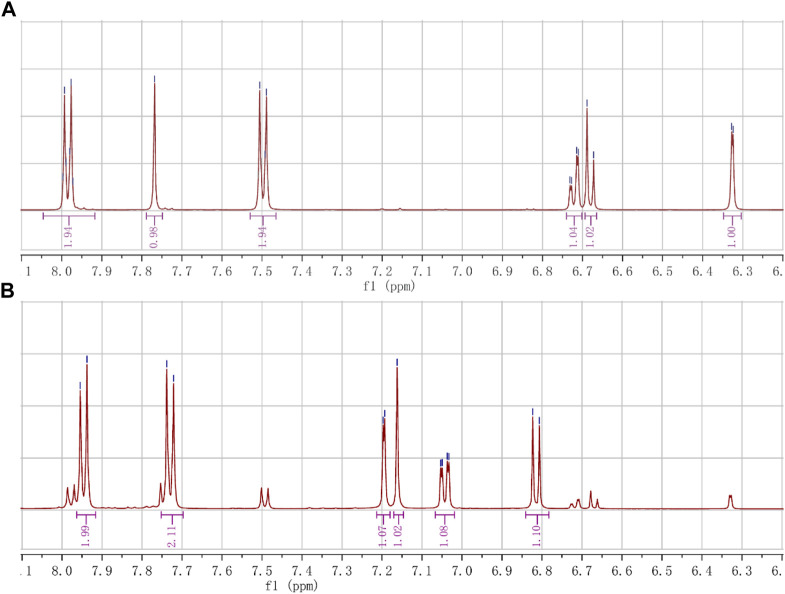
The chemical shifts of hydrogen (C=C-H) of the *cis-trans* isomers. **(A)** The shifts of hydrogen (s, 1H, 7.77 ppm) in the (E)-isomer (A1). **(B)** The shifts of hydrogen (s, 1H, 7.16 ppm) in the (*Z*)-isomer (A6).

The double-bonded hydrogen (C=C-H) of *cis-trans* isomers appeared as an isolated single peak, which can be clearly distinguished from the hydrogen on the phenyl ring. Furthermore, the chemical shifts of double-bonded hydrogen in (E)- and (Z)- isomers were also different enough to distinguish one from the other (Figure 4; Table 2).

**TABLE 2:** Comparison of the hydrogen shifts (C=C-H) in FA derivatives.

Derivatives	The chemical shifts of C=C-H (ppm)	
	(*E*)-Isomer	(*Z*)-Isomer
A1	7.77	
A6		7.16
B1	7.76	
B4		7.16
C1	7.73	
C2		7.17

The double-bonded hydrogen (C=C-H) in the (E)-isomer (A1) appeared at 7.77 ppm as a single peak, while the aromatic hydrogens showed double or quadruple peaks. The symmetrical hydrogens on the aromatic ring with the 4-methanesulfonyl group showed double peaks that appeared at 7.98 ppm (d, J = 8.4 Hz, 2H; 3′, 5′-H) or 7.50 ppm (d, J = 8.3 Hz, 2H; 2′, 6′-H), where the ortho hydrogens (3′, 5′-H) of the methanesulfonyl group moved to higher frequency due to the electron-withdrawing. The hydrogens on the aromatic ring with the phenolic hydroxyl group showed double peaks that appeared at 6.33 ppm (d, J = 2.0 Hz, 1H; 2-H) and 6.68 ppm (d, J = 8.2 Hz, 1H; 5-H), or quadruple peaks that appeared at 6.72 ppm (dd, J = 8.5, 1.9 Hz, 1H; 6-H), of which the 6-H was split into quartet by the ortho-proton (5-H) and meta-proton (2-H).

The double-bonded hydrogen (C=C-H) in the (Z)-isomer (A6) appeared at 7.16 ppm as an isolated single peak, also different from the double or quadruple peaks of the aromatic hydrogens. The symmetrical hydrogens on the aromatic ring with the 4-methanesulfonyl group showed double peaks that appeared at 7.95 ppm (d, J = 8.6 Hz, 2H; 3′, 5′-H) or 7.73 ppm (d, J = 8.6 Hz, 2H; 2′, 6′-H). The hydrogens on the other aromatic ring showed double peaks that appeared at 7.20 ppm (d, J = 2.1 Hz, 1H; 2-H) and 6.81 ppm (d, J = 8.2 Hz, 1H; 5-H), or quadruple peaks that appeared at 7.04 ppm (dd, J = 8.3, 2.0 Hz, 1H; 6-H).

The chemical shifts of the double-bonded hydrogen (C=C-H) appeared at 7.77 ppm (s, 1H; C=C-H) in the (E)-isomer moving to the higher frequency because of the hydrogen being deshielded by the carboxyl group on the same side of the double bond, while appeared at 7.16 ppm (s, 1H; C=C-H) in the (Z)-isomer moving to the lower frequency compared to the (E)-isomer due to the hydrogen being deshielded lowerly by the carboxyl group on the other side of the double bond. The hydrogen in the (E)-isomers, such as A1, B1, and C1, appeared at between 7.7 and 7.9 ppm (DMSO-d6 as the solvent), while it appeared at around 7.1 ppm (DMSO-d6 as the solvent) in the corresponding (Z)-isomers, such as A6, B4, and C2. The A1 was esterified to obtain A2, and further acylated to finally give A5. Therefore, the A5 with a better anti-RSV effect had an isolated single peak at 7.87 ppm (s, 1H), which should also be of the (E)-isomer.

##### 2.1.2.2 Retention times of *cis-trans* isomers in HPLC

The HPLC was performed on a C_18_ reversed-phase column, with methanol and water as the mobile phases, and 0.06%–1% acetic acid was added, and then FA derivatives showed symmetrical peak shapes and good resolution. If FA derivatives became less polar, it led to longer retention times on the C_18_ column, as well as increasing the polarity of the mobile phase, also leading to longer retention times. These derivatives were well resolved under the mobile phase of methanol: water (0.06% acetic acid) = 5:5. The A1 had a retention time of 6.734 min, and A2 derived from esterified A1 and A3 derived from methylated A1 both decreased their polarities with retention times of 12.052 min and 13.884 min, respectively, and A5 further derived from acylated A2 showed less polarity with retention time over 40 min, which was preferable for improving its lipid solubility.

**FIGURE 5:**
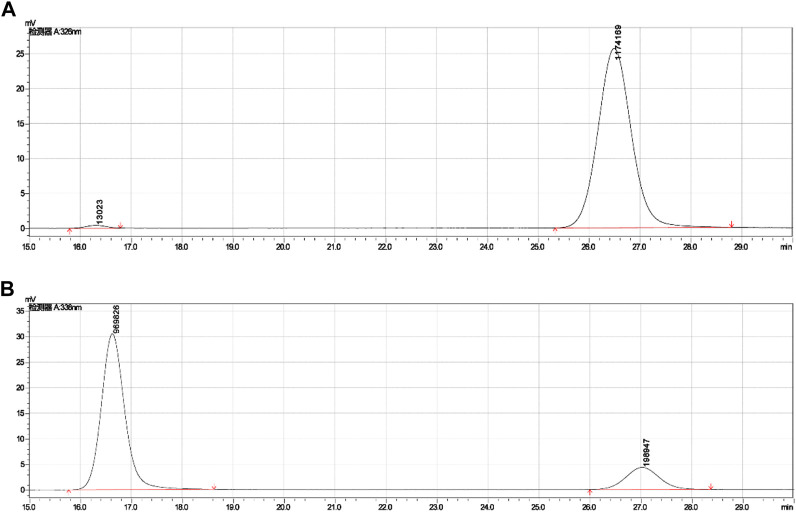
Retention times of *cis-trans* isomers (A1 and A6) in HPLC. **(A)** Retention time (26.483) of (E)-isomer (A1). **(B)** Retention time (16.296) of (*Z*)-isomer (A6).

These derivatives existed *cis-trans* isomers, such as A1 and A6, B1, and B4, and the (Z)-isomer had a smaller retention time and appeared as an earlier peak than (E)-isomer on the reversed-phase column in HPLC, indicating that the (Z)-isomer is more polarized than the (E)-isomer. The (E)-isomer (A1) had a retention time of 26.483 min, and the (Z)-isomer (A6) had a retention time of 16.296 min under the mobile phase of methanol: water (0.1% acetic acid) = 3:7, as shown in Table 3; Figure 5. Other *cis-trans* isomers had similar trends, such as B1 and B4. The polarity of (Z)-isomer is larger than (E)-isomer, and it suggested that the (Z)-isomer had a spreading structure and a stronger conjugation effect because of its two aromatic rings on different sides of the ethylene bridge, and then the electrons of phenolic hydroxyl and carboxyl groups were transferred to the benzene ring, making the (Z)-isomer more polar.

**TABLE 3:** Comparison of retention time of *cis-trans* isomers in HPLC.

Derivatives	Retention time (min)	
	Methanol: Water	Methanol: Water
	(Acetic acid 0.06%) = 5:5	(Acetic acid 1%) = 3:7
A1	6.734	26.483
A6	3.745	16.296
B1	6.252	22.706
B4	3.528	14.119
C1	8.092	—
C2	4.170	—

##### 2.1.2.3 The λ_max_ of *cis-trans* isomers in UV-vis spectroscopy

The A1 had an absorbance maximum (λ_max_) at 326 nm in methanol, and its phenolic hydroxyl group was methylated (A3, λ_max_ = 324 nm) or acylated (A4, λ_max_ = 284 nm) to both appear blue-shifted, and its carboxyl group was esterified (A2, λ_max_ = 330 nm) to appear red-shifted, as well as the A5 (λ_max_ = 286 nm) esterified from A4 also appeared red-shifted compared to A4. Their corresponding sodium salts appeared blue-shifted, such as C6 (λ_max_ = 314 nm, derived from A1), C7 (λ_max_ = 276 nm, derived from A4), and C8 (λ_max_ = 326 nm, derived from A2).

**FIGURE 6:**
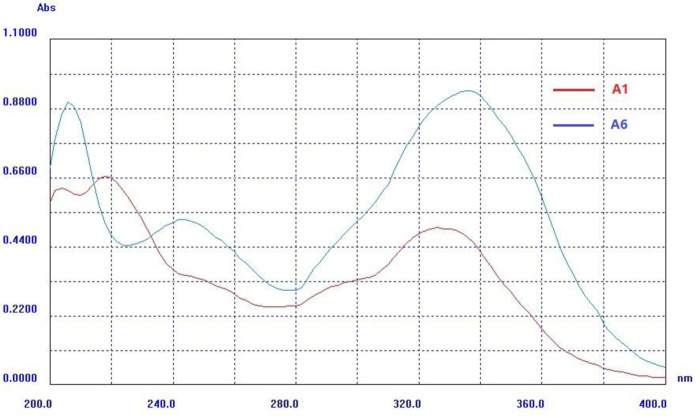
The absorbance maximum (λ_max_) of *cis-trans* isomers (A1 and A6) in UV.

The (Z)-isomer’s absorbance band appeared red-shifted compared with the (E)-isomer, and its absorbance intensity (ε_max_) also obviously increased, as shown in Table 4; Figure 6. For example, the (E)-isomer (A1) had an absorbance band with λ_max_ = 326 nm and *ε* = 17,000, and the (Z)-isomer (A6) with λ_max_ = 336 nm and *ε* = 31,000, as well as other *cis-trans* isomers with similar trends, such as B1 and B4. It is possible that the electrons in the (Z)-isomer transfer from phenolic hydroxyl and carboxyl groups to the benzene ring, making λ_max_ appear red-shifted because the (Z)-isomer had a spreading structure and a stronger conjugation effect when two aromatic rings were on different sides of the ethylene bridge.

**TABLE 4:** Comparison of absorbance spectra of *cis-trans* isomers in UV.

Derivatives	λ_max_ (nm)	ε_max_ (10^∧^4)
A1	326	1.7
A6	336	3.1
B1	310	1.2
B4	320	2.8

### 2.2 Biological experiment

#### 2.2.1 Antiviral activity of FA derivatives

The results of FA derivatives against RSV, HSV-1, and EV71 were shown in Table 1. Ribavirin inhibited RSV, HSV-1, and EV71, but FA had no inhibitory effect. The A1, A2, A5, A6, A7, A8, B1, B2, and B4, which were obtained after modification with FA, had a certain anti-RSV effect (TI ≥ 4). The A2 showed a TI of 16, which was similar to that of ribavirin, while the TI of A5 was 32, which showed the better selective anti-RSV effect than FA and ribavirin. These derivatives did not work on EV71, but some of them had a weak anti-HSV-1 effect (TI = 8).

FA had no inhibitory effect on RSV, but when the benzene ring was introduced into the α-position of the carboxyl group of FA to obtain A7, the inhibitory effect on RSV would be improved, and A1 still certainly inhibited RSV when the 4-methylsulfonyl group was introduced into the α-position phenyl of A7 to obtain A1. The A2 derived from esterified A1 further improved its anti-RSV effect, and the A5 derived from acylated A2 significantly improved its anti-RSV effect. The A3 derived from the methylation of A1 had no inhibitory effect on RSV. The A6 was the (Z)-isomers of A1 with little difference in anti-RSV effect. The B2 and B3 did not show similar antiviral effects because of esterification and acylation compared with A2 and A5, respectively. However, their anti-RSV effect would be obviously reduced after bromine was introduced. In these FA derivatives with diphenyl acrylic acid as the core structure, modified by methylation, acylation, and esterification, *etc*., no obvious rule was found between the structure and anti-RSV activity, but the specific A5 was found to have a selective inhibitory effect on RSV, which was significantly better than FA and ribavirin.

#### 2.2.2 Scavenging effect on free radicals

**FIGURE 7:**
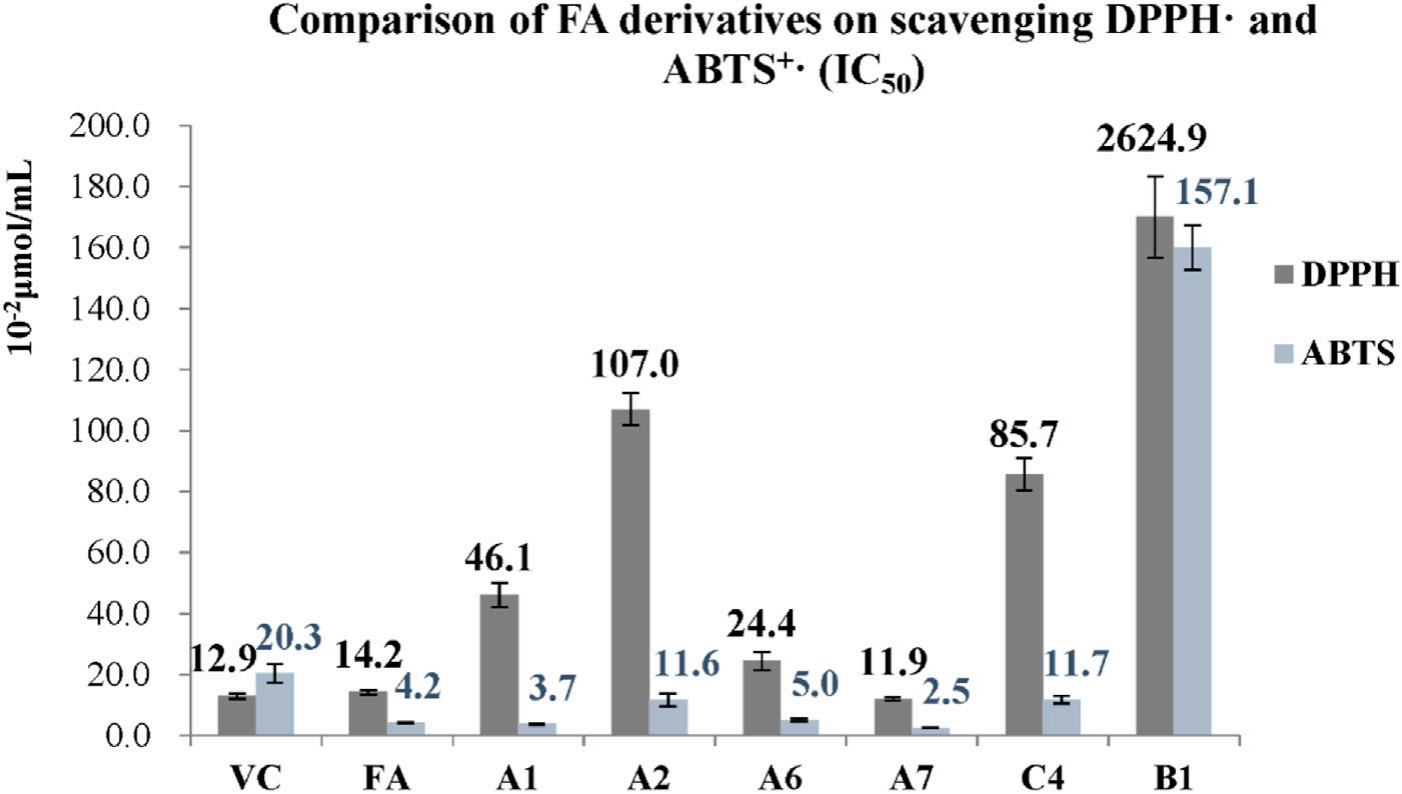
Comparison of FA derivatives on scavenging DPPH· and ABTS^+^ (IC_50_).

The antioxidant effect of FA derivatives was evaluated by the scavenging ability of DPPH· and ABTS^+^· *in vitro*, and measured as the half maximal inhibitory concentration (IC_50_), the results were shown in Figure 7.

The IC_50_ values of vitamin C (Vc) for DPPH· and ABTS^+^· were 0.129 and 0.203 μmol/mL, and FA had similar scavenging effects with IC_50_ values of 0.142 and 0.042 μmol/mL for DPPH and ABTS^+^·, respectively. The scavenging effect of the A1, A2, A6, A7, and C4 on ABTS^+^· was comparable to that of FA, but their effect on DPPH· was weaker, where these derivatives with the IC_50_ values of 0.037, 0.116, 0.050, 0.025, and 0.117 μmol/mL for ABTS^+^· and 0.461, 1.07, 0.244, 0.119, and 0.857 μmol/mL for DPPH, respectively. When the phenolic hydroxyl group was acylated (A4, A5, A9, B3, and C3) or methylated (A3 and A8), their antioxidant activities were not detected. The scavenging effects of A5 disappeared after its phenolic hydroxyl group was acylated, but A5 was easily decomposed to form A2 with better antioxidant effects.

#### 2.2.3 Proliferative effects on cardiovascular cells

**FIGURE 8:**
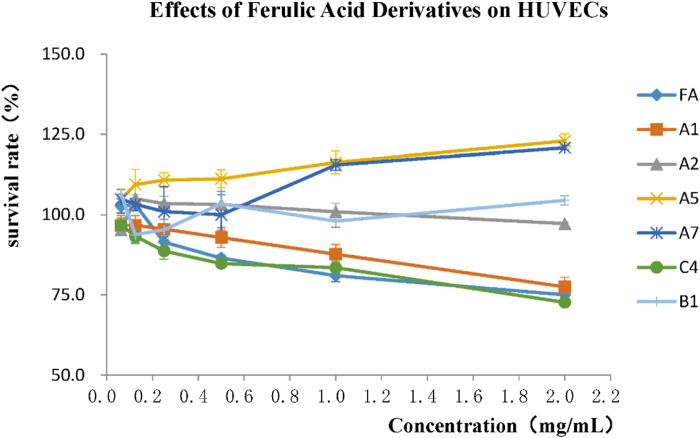
Effects of FA derivatives on proliferation of HUVECs.

The effect of FA derivatives on cardiovascular cells was evaluated by their proliferative activities on human umbilical vein endothelial cells (HUVECs), and the cell survival rates of FA, A1, A2, A5, A7, C4, and B1 were detected by MTT assay as shown in Figure 8. FA had a proliferative effect on HUVECs at a concentration of 0.2 mg/mL or below, and the survival rate of HUVECs gradually decreased with increasing FA concentration. The A1 and C4 showed a similar trend with FA. The survival rates of A2, A5 and B1 on HUVECs were above 90%, especially A5 had an obvious proliferative effect on HUVECs, which was beneficial to cardiovascular cells.

## 3 Materials and methods

### 3.1 Instruments and reagents

^1^H NMR (solvent DMSO-d_6_) spectral analyses were recorded on Agilent 400 MHz and 500 MHz spectrometer (Agilent Technologies Co., Ltd.) and chemical shifts were quoted in *δ* as parts per million (ppm) downfield with tetramethylsilane (TMS) as internal standard. Coupling constants, J, are expressed in hertz (Hz). MS data were recorded on Applied Biosystems API4000, IR spectra were recorded on a Nicolet NEXUS 470 spectrometer with KBr pellets. HPLC (Detector SPD-10A, Injector SIL-10ADVP) was tested using LC-10Atvp spectrometer (Shimadzu Corporation, Japan). Column: 150 mm × 4.6 mm prefilled with ZORBAX SB-C18, 5 μm (Agilent Technologies Co., Ltd.). UV-Vis spectra were recorded on a UV9100B spectrometer (Beijing Labtech Instrument Co., Ltd.). Full-wavelength enzyme labeler was determined on MultiskanGo-1510 and MK3 instrument (China Semel Faisel Technology Co., Ltd.).

Hep2 was RSV, HSV-1 sensitive cells; MA104 was EV71 sensitive cells; HUVECs were obtained from the Chinese Academy of Sciences Cell Bank. RSV long strain virus was quoted from the Poisonous Seed Room of the Institute of Virology, Chinese Academy of Preventive Medicine; HSV-1 F strain virus was quoted from the Microbiology Teaching and Research Office of Shandong Medical University; EV71 was isolated from patients with hand, foot and mouth disease in Jinan Infectious Diseases Hospital. The above cells and viruses were stored in the University Antiviral Center.

### 3.2 Chemical experiment

#### 3.2.1 General Process of Preparation of Derivatives

4-methanesulfonylphenylacetic acid (0.01 mol) and 3-methoxy-4-hydroxybenzaldehyde (0.01 mol), which were mixed in freshly distilled acetic anhydride (6.0 mL) and triethylamine (0.7 mL) for 5 h at 130°C–140°C, cooled down to 100°C, and then water (1–2 mL) was added, stirred for 5 min, cooled to room temperature, poured into water (60 mL), left at room temperature, and filtered to obtain a solid. The solid was treated with NaOH until pH 12 was reached, stirred at 90°C for 2 h, cooled to room temperature, dilute HCl was added to reach pH 6-7, and a white-like solid was obtained. After filtration, the (E)-isomer A1 was obtained by recrystallization with acetic acid.

The prepared A1 was suspended in anhydrous ethanol, then added to concentrated sulfuric acid and refluxed for 6 h. The esterified product was precipitated and recrystallized with acetic acid to obtain A2 as an (E)-isomer. The A2 was added to acetic anhydride, refluxed for 3 h, and heated to remove excess acetic anhydride, and then recrystallized with acetic acid to obtain the acylated and esterified A5 as an (E)-isomer. Carboxylate C6 was obtained by lyophilizing A1 with an equimolar NaHCO_3_ solution.

The (E)-isomers were mixed with a small amount of (Z)-isomers, and the purity of the (E)-isomer can be improved by repeated recrystallization with acetic acid. The solution after precipitating the A1 was adjusted to pH 2-3 with dilute HCl, and a small amount of solid was precipitated to obtain A6 as an (Z)-isomer. The (E)-isomer can be removed from the (Z)-isomer with repeated alkali-solution and acid-isolation, and finally the pure (Z)-isomer was obtained.

The synthesis of other compounds, and the separation of *cis-trans* isomers were similar to the reaction processes described above.

#### 3.2.2 Synthesis and characterization

##### 3.2.2.1 (E)-2-(4-Methanesulfonylphenyl)-3-(3-methoxy-4-hydroxyphenyl)acrylic acid (A1)

4-methanesulfonylphenylacetic acid and 3-methoxy-4-hydroxybenzaldehyde were mixed and reacted according to the conditions described in “3.2.1 General Process of Preparation of Derivatives” to obtain a white solid A1, yield 62%, m.p. 235.2°C–235.3°C; HPLC (methanol: water = 5:5, 0.06% acetic acid): retention time 6.734 min, content 98.86%; UV, λ_max_ = 326 nm (MeOH).

^1^H NMR (500 MHz, DMSO-d_6_) *δ* 12.32 (s, 1H; -COOH), 9.56 (s, 1H; -OH), 7.98 (d, J = 8.4 Hz, 2H), 7.77 (s, 1H; C=CH), 7.50 (d, J = 8.3 Hz, 2H), 6.72 (dd, J = 8.5, 1.9 Hz, 1H), 6.68 (d, J = 8.2 Hz, 1H), 6.33 (d, J = 2.0 Hz, 1H), 3.30 (s, 3H; -OCH_3_), 3.24 (s, 3H; -SO_2_CH_3_); ^13^C NMR (126 MHz, DMSO-d_6_) *δ* 168.07, 148.59, 147.16, 143.08, 140.81, 139.94, 131.11, 128.30, 127.45, 125.81, 125.11, 115.51, 113.12, 54.73, 43.56.

##### 3.2.2.2 Ethyl (E)-2-(4-methanesulfonylphenyl)-3-(3-methoxy-4-hydroxyphenyl) acrylate (A2)

The previous A1 was added to anhydrous ethanol and refluxed with concentrated sulfuric acid, the white solid A2 was obtained according to the above conditions, yield 74%, m.p. 133.3°C–133.6°C; HPLC (methanol: water = 5:5, 0.06% acetic acid): retention time 12.052 min, content 97.0%; UV, λ_max_ = 330 nm (MeOH).

^1^H NMR (500 MHz, DMSO-d_6_) *δ* 9.62 (s, 1H), 7.98 (dt, J = 8.5, 1.4 Hz, 2H), 7.77 (s, 1H), 7.50 (dt, J = 8.5, 1.7 Hz, 2H), 6.72 (dt, J = 8.3, 1.9 Hz, 1H), 6.66 (dt, J = 8.2, 1.8 Hz, 1H), 6.31 (d, J = 1.8 Hz, 1H), 4.18 (dd, J = 7.1, 1.1 Hz, 2H), 3.29 (s, 3H), 3.25 (s, 3H), 1.20 (t, J = 7.1 Hz, 3H); ^13^C NMR (126 MHz, DMSO-d_6_) *δ* 166.75, 149.12, 147.48, 142.78, 141.56, 140.46, 131.44, 128.00, 127.83, 127.75, 126.75, 126.29, 125.13, 115.81, 113.49, 61.19, 55.07, 43.80, 14.64; IR (KBr, cm^-1^): 3349.68, 3021.79, 2983.17, 2934.22, 1676.57, 1600.86, 1515.52, 1465.36, 1430.85, 1392.46, 1373.18, 1308.22, 1259.93, 1210.27, 1179.96, 1149.06, 1086.97, 955.16, 831.65, 825.58, 1045.91; ESI-MS (m/z): calcd. is 376.42, found 377.4 [M + H]^+^ for ^C19H20O6S^.

##### 3.2.2.3 (E)-2-(4-Methanesulfonylphenyl)-3-(3,4-dimethoxyphenyl)acrylic acid (A3)

4-methanesulfonylphenylacetic acid and 3,4-dimethoxybenzaldehyde were mixed and reacted according to the conditions described in “3.2.1 General Process of Preparation of Derivatives” to finally obtain a white solid A3, yield 36.4%, m.p. 236.5°C–236.5°C; HPLC (methanol: water = 5:5, 0.06% acetic acid): retention time 13.884 min, content 97.3%; UV, λ_max_ = 324 nm (MeOH).

^1^H NMR (500 MHz, DMSO-d_6_) *δ* 12.49 (s, -OH), 7.97 (d, J = 8.3 Hz, 2H), 7.78 (s, 1H), 7.49 (d, J = 8.3 Hz, 2H), 6.86 (d, J = 8.4 Hz, 1H), 6.82 (d, J = 2.0 Hz, 1H), 6.37 (d, J = 2.0 Hz, 1H), 3.71 (s, 3H), 3.29 (s, 3H), 3.24 (s, 3H); ^13^C NMR (126 MHz, DMSO-d_6_) δ168.23, 150.47, 148.42, 143.10, 140.70, 140.32, 131.33, 129.61, 127.76, 126.66, 125.50, 112.96, 112.94, 111.84, 55.91, 55.90, 55.00, 43.86.

##### 3.2.2.4 (E)-2-(4-Methanesulfonylphenyl)-3-(3-methoxy-4-acetoxyphenyl)acrylic acid (A4)

The A1 was added to acetic anhydride, refluxed, and treated according to the above conditions to obtain a white solid A4, yield 72%, m.p. 228.0°C–229.2°C; HPLC (methanol: water = 5:5, 0.06% acetic acid): retention time 17.730 min, content 98.0%; UV, λ_max_ = 284 nm (MeOH).

^1^H NMR (500 MHz, DMSO-d_6_) *δ* 12.85 (s, 1H), 7.97 (d, J = 8.3 Hz, 2H), 7.84 (s, 1H), 7.50 (d, J = 8.4 Hz, 2H), 6.99 (d, J = 8.2 Hz, 1H), 6.77 (dd, J = 8.3, 1.9 Hz, 1H), 6.61 (d, J = 1.9 Hz, 1H), 3.36 (s, 3H), 3.24 (s, 3H), 2.20 (s, 3H); ^13^C NMR (126 MHz, DMSO-d_6_) *δ* 168.41, 167.69, 150.43, 142.18, 140.21, 140.14, 139.55, 132.69, 132.03, 130.88, 127.44, 123.82, 123.13, 113.97, 55.22, 43.53, 20.46; IR (KBr, cm^-1^): 3434.61, 2623.64, 3025.15, 2975.14, 2941.93, 1771.40, 1675.53, 1611.50, 1595.86, 1508.27, 1465.43, 1451.94, 1416.83, 1389.63, 1296.78, 1256.79, 1195.40, 1155.88, 1090.74, 1024.49, 957.92, 832.55; ESI-MS (m/z): calcd. is 390.41, found 391.3 [M + H]^+^ for ^C19H18O7S^.

##### 3.2.2.5 Ethyl (E)-2-(4-methanesulfonylphenyl)-3-(3-methoxy-4-acetoxyphenyl) acrylate (A5)

The previous A2 was added to acetic anhydride, refluxed, and treated according to the above conditions to obtain a white solid A5, yield 78%, m.p. 133.3°C–133.6°C; HPLC (methanol: water = 6:4): retention time 11.282 min, content 96.9%; UV, λ_max_ = 286 nm (MeOH).

^1^H NMR (500 MHz, DMSO-d_6_) *δ* 7.99 (d, J = 8.3 Hz, 2H), 7.87 (s, 1H), 7.53 (d, J = 8.3 Hz, 2H), 7.00 (d, J = 8.2 Hz, 1H), 6.78 (dd, J = 8.3, 2.0 Hz, 1H), 6.62 (d, J = 2.0 Hz, 1H), 4.22 (q, J = 7.1 Hz, 2H), 3.37 (s, 3H), 3.26 (s, 3H), 2.20 (s, 3H), 1.23 (t, J = 7.0 Hz, 3H); ^13^C NMR (126 MHz, DMSO-d_6_) *δ* 168.69, 166.44, 150.77, 141.81, 140.75, 140.61, 140.43, 132.76, 131.48, 131.27, 127.83, 124.26, 123.49, 114.38, 61.56, 55.58, 43.77, 20.79, 14.60.

##### 3.2.2.6 (Z)-2-(4-Methanesulfonylphenyl)-3-(3-methoxy-4-hydroxyphenyl)acrylic acid (A6)

In the solution after precipitation of A1, the combined aqueous solutions were treated with dilute HCl until the pH about 2-3 reached, and a small amount of white solid A6 was finally precipitated, yield 6%, m.p. 215.7°C–219.2°C; HPLC (methanol: water = 5:5, 0.06% acetic acid): retention time 2.231 min, content 97.08%; UV, λ_max_ = 336 nm (MeOH).

^1^H NMR (500 MHz, DMSO-d_6_) *δ* 13.42 (s, 1H; -COOH), 9.48 (s, 1H; -OH), 7.95 (d, J = 8.6 Hz, 2H), 7.73 (d, J = 8.6 Hz, 2H), 7.20 (d, J = 2.1 Hz, 1H), 7.16 (s, 1H; C=CH), 7.04 (dd, J = 8.3, 2.0 Hz, 1H), 6.81 (d, J = 8.2 Hz, 1H), 3.78 (s, 3H), 3.23 (s, 3H); ^13^C NMR (126 MHz, DMSO-d_6_) *δ* 171.12 (3-C), 148.20, 147.88, 142.29, 139.84, 131.39, 131.36 (1-C), 127.98 (9,11-C), 126.70 (8,12-C), 126.66, 122.90 (13-C), 116.02 (14-C), 112.98 (17-C), 55.97 (24-C), 44.04 (22-C).

##### 3.2.2.7 (E)-2-Phenyl-3-(3-methoxy-4-hydroxyphenyl)acrylic acid (A7)

Phenylacetic acid and 3-methoxy-4-hydroxybenzaldehyde were mixed and reacted according to the above conditions to finally obtain a white solid A7, yield 23.0%, m.p. 191.6°C–191.9°C; HPLC (methanol: water = 5:5, 0.06% acetic acid): retention time 8.072 min, content 97.1%; UV, λ_max_ = 310 nm (MeOH).

^1^H NMR (500 MHz, DMSO-d_6_) *δ* 12.41 (s, 1H), 9.45 (s, 1H), 7.66 (s, 1H), 7.42 (t, J = 7.4 Hz, 2H), 7.35 (t, J = 7.4 Hz, 1H), 7.19 (d, J = 6.9 Hz, 2H), 6.68 (dd, J = 8.3, 2.0 Hz, 1H), 6.63 (d, J = 8.2 Hz, 1H), 6.42 (d, J = 2.0 Hz, 1H), 3.30 (s, 3H); ^13^C NMR (126 MHz, DMSO-d_6_) *δ* 169.02, 148.53, 147.36, 139.96, 137.59, 130.12, 129.08, 127.78, 126.00, 125.81, 115.60, 113.59, 55.10.

##### 3.2.2.8 (E)-2-Phenyl-3-(3,4-dimethoxyphenyl)acrylic acid (A8)

Phenylacetic acid and 3,4-dimethoxybenzaldehyde were mixed and reacted according to the conditions described in “3.2.1 General Process of Preparation of Derivatives” to finally obtain a white solid A8, yield 22.8%, m.p. 229.0°C–229.1°C; HPLC (methanol: water = 6:4): retention time 6.094 min, content 95.8%; UV, λ_max_ = 308 nm (MeOH).

^1^H NMR (400 MHz, DMSO-d_6_) *δ* 12.52 (s, 1H), 7.72 (s, 1H), 7.43 (d, J = 7.3 Hz, 2H), 7.37 (t, J = 7.3 Hz, 1H), 7.21 (d, J = 7.3 Hz, 2H), 6.90 – 6.76 (m, 2H), 6.46 (d, J = 1.6 Hz, 1H), 3.72 (s, 3H), 3.30 (s, 3H); ^13^C NMR (101 MHz, DMSO-d_6_) *δ* 168.45, 149.69, 147.83, 139.13, 136.92, 130.65, 129.59, 128.62, 127.38, 126.81, 124.83, 112.50, 111.17, 55.37, 54.50.

##### 3.2.2.9 (E)-2-Phenyl-3-(3-methoxy-4-acetoxyphenyl)acrylic acid (A9)

The A7 was added to acetic anhydride, refluxed, and treated according to the above conditions to obtain a white solid A9, yield 18.5%, m.p. 209.5°C–209.9°C; HPLC (methanol: water = 5:5, 0.06% acetic acid): retention time 19.271 min, content 98.9%; UV, λ_max_ = 284 nm (MeOH).

^1^H NMR (500 MHz, DMSO-d_6_) *δ* 12.60 (s, 1H), 7.75 (s, 1H), 7.42 (d, J = 7.6 Hz, 2H), 7.37 (d, J = 7.0 Hz, 1H), 7.21 (d, J = 6.9 Hz, 2H), 6.95 (dd, J = 8.2 Hz, 1H), 6.78 (dd, J = 8.3, 1.9 Hz, 1H), 6.66 (d, J = 1.9 Hz, 1H), 3.36 (s, 3H), 2.20 (s, 3H); ^13^C NMR (126 MHz, DMSO-d_6_) *δ* 168.73, 168.67, 150.62, 140.19, 138.71, 136.85, 133.77, 133.56, 129.91, 129.14, 128.08, 124.06, 123.19, 114.23, 55.50, 21.50, 20.79.

##### 3.2.2.10 (E)-2-(4-Methanesulfonylphenyl)-3-(4-hydroxyphenyl)acrylic acid (B1)

4-Methylsulfonylphenylacetic acid and p-hydroxybenzaldehyde were mixed and reacted according to the above conditions to finally obtain a white solid B1, yield 68%, m.p. 236.9°C–237.7°C; HPLC (methanol: water = 5:5, 0.06% acetic acid): retention time 6.252 min, content 98.86%; UV, λ_max_ = 310 nm (MeOH).

^1^H NMR (500 MHz, DMSO-d_6_) *δ* 12.34 (s, 1H; -COOH), 9.93 (s, 1H; -OH), 7.94 (d, J = 8.4 Hz, 2H), 7.76 (s, 1H; -C=C-H), 7.46 (d, J = 8.4 Hz, 2H), 6.89 (d, J = 8.8 Hz, 2H), 6.60 (d, J = 8.7 Hz, 2H), 3.27 (s, 3H); ^13^C NMR (126 MHz, DMSO-d_6_) *δ* 168.40, 159.34, 143.03, 140.86, 140.14, 132.80, 131.24, 128.56, 127.63, 125.07, 115.88, 43.99.

##### 3.2.2.11 Ethyl (E)-2-(4-methanesulfonylphenyl)-3-(4-hydroxyphenyl)acrylate (B2)

The previous B1 was added to anhydrous ethanol and refluxed with concentrated sulfuric acid, the white solid B2 was obtained according to the above conditions, yield 78.5%, m.p. 151.6°C–152.1°C; HPLC (methanol: water = 5:5, 0.06% acetic acid): retention time 13.884 min, content 99.2%.

^1^H NMR (500 MHz, DMSO-d_6_) *δ* 10.01 (s, 1H), 8.01 – 7.89 (m, 2H), 7.79 (s, 1H), 7.54 – 7.41 (m, 2H), 6.95 – 6.83 (m, 2H), 6.67 – 6.56 (m, 2H), 4.18 (q, *J* = 7.0 Hz, 2H), 3.29 (s, 3H), 1.21 (t, J = 7.1 Hz, 3H); ^13^C NMR (126 MHz, DMSO-d_6_) *δ* 166.35, 159.06, 141.92, 140.91, 139.88, 132.47, 130.82, 127.28, 127.23, 124.36, 115.44, 60.72, 43.44, 14.15.

##### 3.2.2.12 Ethyl (E)-2-(4-methanesulfonylphenyl)-3-(4-acetoxyphenyl)acrylate (B3)

The previous B2 was added to acetic anhydride, refluxed, and treated according to the above conditions to obtain a white flocculent solid B3, yield 82.4%, m.p. 151.6°C–152.1°C; HPLC (methanol: water = 5:5, 0.06% acetic acid): retention time 17.328 min, content 98.6%.

^1^H NMR (500 MHz, DMSO-d_6_) *δ* 7.95 (d, J = 8.1 Hz, 2H), 7.89 (s, 1H), 7.50 (d, J = 8.4 Hz, 2H), 7.09 (d, J = 8.8 Hz, 2H), 7.02 (d, J = 8.9 Hz, 2H), 4.21 (d, J = 7.1 Hz, 2H), 3.30 (s, 3H), 2.24 (s, 3H), 1.23 (t, J = 8.5, 6.3 Hz, 3H); ^13^C NMR (126 MHz, DMSO-d_6_) *δ* 169.33, 166.50, 151.56, 141.51, 140.66, 140.27, 132.00, 131.68, 131.48, 131.23, 127.71, 122.48, 61.56, 43.91, 21.27, 14.58.

##### 3.2.2.13 (Z)-2-(4-Methanesulfonylphenyl)-3-(4-hydroxyphenyl)acrylic acid (B4)

The pH of the solution after precipitation of B1 was adjusted to about 2-3 with dilute HCl, and a small amount of white solid B4 was finally precipitated, yield 7%, m.p. 226.8°C–228.0°C; HPLC (methanol: water = 5:5, 0.06% acetic acid): retention time 3.528 min, content 97.08%; UV, λ_max_ = 320 nm (MeOH).

^1^H NMR (500 MHz, DMSO-d_6_) *δ* 13.47 (s, 1H; -COOH), 9.85 (s, 1H), 7.93 (d, J = 6.7 Hz, 2H), 7.72 (d, J = 6.7 Hz, 2H), 7.41 (d, J = 4.5 Hz, 2H), 7.16 (s, 1H; -C=CH), 6.80 (d, J = 5.1 Hz, 2H), 3.22 (s, 3H; -SO_2_CH_3_); ^13^C NMR (126 MHz, DMSO-d_6_) *δ* 170.97, 158.75, 142.33, 139.83, 131.34, 131.12, 130.80, 127.95, 126.74, 126.23, 115.95, 44.04.

##### 3.2.2.14 (E)-2-(4-Methanesulfonylphenyl)-3-(3-bromo-4-hydroxyphenyl)acrylic acid (C1)

4-Methylsulfonylphenylacetic acid and 3-bromo-4-hydroxybenzaldehyde were mixed and reacted according to the conditions described in “3.2.1 General Process of Preparation of Derivatives” to finally obtain a white solid C1, yield 52%, m.p. 234.9°C–236.2°C; HPLC (methanol: water = 5:5, 0.06% acetic acid): retention time 8.092 min, content 96.50%.

^1^H NMR (500 MHz, DMSO-d_6_) *δ* 12.79 (s, 1H), 10.76 (s, 1H), 7.95 (d, J = 8.4 Hz, 2H), 7.73 (s, 1H; C=CH), 7.46 (d, J = 8.4 Hz, 2H), 7.10 (d, J = 2.1 Hz, 1H), 6.88 (dd, J = 8.6, 2.2 Hz, 1H), 6.77 (d, J = 8.5 Hz, 1H), 3.25 (s, 3H); ^13^C NMR (126 MHz, DMSO-d_6_) *δ* 168.08, 155.75, 142.57, 140.38, 139.36, 135.44, 131.75, 131.16, 130.20, 127.72, 126.79, 116.51, 109.76, 44.11.

##### 3.2.2.15 (Z)-2-(4-Methanesulfonylphenyl)-3-(3-bromo-4-hydroxyphenyl)acrylic acid (C2)

The pH of the solution after precipitation of C1 was adjusted to about 2-3 with dilute HCl, and a small amount of white solid C2 was finally precipitated, yield 10%, m.p. 223.4°C–227.7°C; HPLC (methanol: water = 5:5, 0.06% acetic acid): retention time 4.170 min, content 95.4%.

^1^H NMR (500 MHz, DMSO-d_6_) *δ* 13.65 (s, 1H), 10.72 (s, 1H), 7.95 (d, J = 8.5 Hz, 2H), 7.74 (s, 1H), 7.73 (d, J = 6.4 Hz, 2H), 7.40 (dd, J = 8.6, 2.2 Hz, 1H), 7.17 (s, 1H; C=CH), 7.00 (d, J = 8.5 Hz, 1H), 3.23 (s, 3H); ^13^C NMR (126 MHz, DMSO-d_6_) *δ* 170.66, 155.24, 141.94, 140.15, 133.38, 132.50, 129.89, 129.87, 127.99, 127.97, 126.94, 116.80, 109.94, 44.02.

##### 3.2.2.16 (E)-2-(4-Methanesulfonylphenyl)-3-(3-bromo-4-acetoxyphenyl)acrylic acid (C3)

The previous C1 was added to acetic anhydride, refluxed, and treated according to the above conditions to obtain a white solid C3, yield 36.0%, m.p. 210.1°C–212.0°C; HPLC (methanol: water = 5:5, 0.06% acetic acid): retention time 16.893 min, content 96.7%.

^1^H NMR (500 MHz, DMSO-d_6_) *δ* 12.43 (s, 3H), 7.96 (d, J = 8.4 Hz, 2H), 7.84 (s, 1H), 7.49 (d, J = 8.4 Hz, 2H), 7.35 (d, J = 2.1 Hz, 1H), 7.15 (d, J = 8.4 Hz, 1H), 7.05 (dd, J = 8.5, 2.1 Hz, 1H), 3.25 (s, 3H), 2.28 (s, 3H); ^13^C NMR (126 MHz, DMSO-d_6_) *δ* 168.54, 167.76, 148.62, 141.77, 140.64, 138.27, 134.98, 134.13, 133.72, 131.11, 131.07, 127.72, 124.60, 116.13, 44.05.

##### 3.2.2.17 (E)-2-(4-Methanesulfonylphenyl)-3-(3-methoxy-4-hydroxy-5-bromophenyl)acrylic acid (C4)

4-Methylsulfonylphenylacetic acid and 3-methoxy-4-hydroxy-5-bromobenzalde-hyde were mixed and reacted according to the conditions described in “3.2.1 General Process of Preparation of Derivatives” to finally obtain a white solid C4, yield 50%, m.p. 232.0°C–234.2°C; HPLC (methanol: water = 5:5, 0.06% acetic acid): retention time 10.093 min, content 98.4%.

^1^H NMR (500 MHz, DMSO-d_6_) *δ* 12.56 (s, 1H), 10.01 (s, 1H), 7.99 (dd, J = 8.4, 3.7 Hz, 2H), 7.74 (s, 1H), 7.50 (dd, J = 8.4, 3.7 Hz, 2H), 6.96 (d, J = 3.6 Hz, 1H), 6.38 (d, J = 3.4 Hz, 1H), 3.38 (s, 3H), 3.25 (s, 3H); ^13^C NMR (126 MHz, DMSO-d_6_) *δ* 168.03, 148.01, 145.66, 142.83, 140.46, 139.65, 131.30, 130.44, 128.40, 127.81, 126.27, 112.64, 109.54, 55.84, 43.95,21.52.

##### 3.2.2.18 (E)-2-(4-Methanesulfonylphenyl)-3-(3-methoxy-4-acetoxy-5-bromophenyl) acrylic acid (C5)

The previous C4 was added to acetic anhydride, refluxed, and treated according to the above conditions to obtain a white solid C5, yield 7%, m.p. 216.4°C–218.3°C; HPLC (methanol: water = 5:5, 0.06% acetic acid): retention time 26.183 min, content 97.5%.

^1^H NMR (500 MHz, DMSO-d_6_) *δ* 13.08 (s, 1H), 8.26 (d, J = 8.9 Hz, 1H), 8.09 (s, 1H), 7.79 (d, J = 8.8 Hz, 2H), 7.31 (t, J = 2.1 Hz, 1H), 6.91 (t, J = 2.1 Hz, 1H), 3.66 (s, 3H), 3.51 (s, 3H), 2.54 (s, 3H); ^13^C NMR (126 MHz, DMSO-d_6_) *δ* 168.03, 167.94, 142.26, 140.94, 138.77, 138.26, 134.31, 134.01, 131.36, 128.01, 127.01, 117.13, 114.07, 56.36, 44.12,20.74.

##### 3.2.2.19 Sodium (E)-2-(4-methanesulfonylphenyl)-3-(3-methoxy-4-hydroxyphenyl) acrylate (C6)

The A1 was reacted with equimolar NaHCO_3_ and lyophilized *in vacuo* to obtain a bright yellow solid C6, m.p. 150 °C, this moment it became foam; UV, A1: λ_max_ = 326 nm, C6: λ_max_ = 314 nm (MeOH), with a blue shift.

^1^H NMR (400 MHz, DMSO-d_6_) *δ* 7.91 (d, J = 8.4 Hz, 2H), 7.80 (dd, J = 9.4 Hz, 1H), 7.58 (s, 1H), 7.43 (dd, J = 8.3, 2H), 6.71 (dd, J = 8.2 Hz, 1H), 6.64 (d, J = 8.2, 1.4 Hz, 1H), 6.27 (d, J = 1.9 Hz, 1H), 3.28 (s, 3H), 3.22 (s, 3H).

##### 3.2.2.20 Sodium (E)-2-(4-methanesulfonylphenyl)-3-(3-methoxy-4-acetoxyphenyl) acrylate (C7)

The A4 was reacted with equimolar NaHCO_3_ and lyophilized *in vacuo* to obtain a white solid C7. UV, A4: λ_max_ = 284 nm, C7: λ_max_ = 276 nm (MeOH), with a blue shift.

^1^H NMR (400 MHz, DMSO-d_6_) *δ* 7.85 (d, J = 7.4 Hz, 2H), 7.45 (s, 1H), 7.37 (d, J = 7.4 Hz, 2H), 6.90 (dd, J = 8.2, 1.1 Hz, 1H), 6.64 (d, J = 7.9 Hz, 1H), 6.48 (s, 1H), 3.34 (s, 2H), 3.20 (s, 3H), 2.20 (s, 3H).

##### 3.2.2.21 Sodium (E)-2-(4-methanesulfonylphenyl)-3-(3-methoxy-4-hydroxyphenyl) ethyl acrylate (C8)

The A2 was reacted with equimolar NaOH, heated and lyophilized *in vacuo* to obtain a yellow solid C8. UV, A2: λ_max_ = 330 nm, C8: λ_max_ = 326 nm (MeOH), with a blue shift.

^1^H NMR (400 MHz, DMSO-d_6_) *δ* 7.98 (dd, J = 8.3, 1.4 Hz, 2H), 7.73 (s, 1H), 7.50 (dd, J = 8.2, 1.4 Hz, 2H), 6.69 (d, J = 8.3 Hz, 1H), 6.56 (d, J = 8.3 Hz, 1H), 6.18 (d, J = 2.2 Hz, 1H), 4.16 (dd, J = 7.1, 1.3 Hz, 2H), 3.26 (s, 3H), 3.22 (s, 3H), 1.20 (t, J = 7.1, 1.3 Hz, 3H).

### 3.3 Biological experiment

#### 3.3.1 Antiviral activity assays

The antiviral effect of FA derivatives *in vitro* was evaluated by detecting the inhibitory effect on RSV, HSV-1, and EV71 (Yue et al., 2018).

Ferulic acid and its derivatives were 2-fold serially diluted to 12 concentrations, and Hep2 and MA104 cells were pretreated with these test drugs and ribavirin as control, repeated 3 times in parallel. The cytopathic effect (CPE) was observed daily using a microscope, and cytotoxicity was considered when the CPE was greater than 50%, then the median toxic concentration (TC_50_) and the maximum non-toxic concentration (TC_0_) were obtained. These test drugs were 2-fold serially diluted to 12 concentrations from a starting concentration (TC_0_), and Hep2 and MA104 cells were pretreated with these test drugs and ribavirin as control, repeated 3 times in parallel. RSV and HSV-1 viruses were inoculated on Hep2 cells, and EV71 virus was inoculated on MA104 cells. The cytopathic effect (CPE) was observed daily using a microscope, and recorded when the CPE reached 90%. The 50% infective concentration of CPE was considered as the median effective concentration (EC_50_). The TI of FA derivatives was calculated according to the following Eq. 1, and those with TI > 4 were judged as effective.

$$TI=\frac{{TC}_{50}}{{EC}_{50}}$$ (1)

#### 3.3.2 Free radical scavenging assays

The scavenging ability of FA derivatives to free radicals was modified according to the literature (Zeng and Shi, 2013). The scavenging ability of FA derivatives on DPPH· and ABTS^+^ was evaluated by the IC_50_ values, and Vc was used as control. As the IC_50_ value was smaller, the scavenging ability was greater.

Ferulic acid and its derivatives were diluted to six concentrations, 50 μL of these test drugs were added to the 96-well plate, and then 100 μL of DPPH· (120 μg/mL) was added to it, repeated 3 times in parallel. The solutions were mixed and kept in the dark for 30 min, and the absorbance (517 nm) was measured to calculate the scavenging rate of DPPH· according to Eq. 2. 50 μL of these test drugs was added to the 96-well plate, and then 100 μL of ABTS^+^· (135 μg/mL) were added to it, repeated 3 times in parallel. The solutions were mixed and kept in the dark for 6 min, and the absorbance (734 nm) was measured to calculate the scavenging rate of ABTS^+^· according to Eq. 2. The IC_50_ value was calculated with the concentration as the abscissa and the scavenging rate as the ordinate.

$$Scavenging\;rate\%=\frac{{OD}_{blank\;controls}{-OD}_{drug\;groups}}{{OD}_{blank\;controls}}\times 100\%$$ (2)

#### 3.3.3 Effects on cardiovascular cells

The activity of FA derivatives on cardiomyocytes was evaluated by the effect on the proliferation of HUVECs. Ferulic acid and its derivatives were diluted to six concentrations. HUVECs in the 96-well plates were divided into three groups: negative controls, blank controls, and drug groups, repeated 3 times in parallel. HUVECs were treated with the test drugs, and cultured for 24 h, and then the Cell viability was assessed by the MTT assay. The absorbance (490 nm) was measured to calculate the cell viability according to Eq. 3:

$$Cell\;viability\%=\frac{{OD}_{drug\;groups}{-OD}_{blank\;controls}}{{OD}_{negative\;controls}{-OD}_{blank\;controls}}\times 100\%$$ (3)

## 4 Conclusion

In the present study, a series of FA derivatives were synthesized and characterized by MS, IR, ^1^H NMR and ^13^C NMR. There were *cis-trans* isomerism related to diphenyl acrylic acid in these derivatives, such as A1 and A6, B1 and B4, C1, and C2, which served as *cis-trans* isomer for each other and were confirmed by ^1^H NMR, HPLC, and UV-vis spectra for the first time. In the ^1^H NMR analysis, the double-bonded hydrogen (C=C-H) of the *cis-trans* isomer appeared as an isolated single peak that distinguished from the aromatic hydrogens, and its chemical shift moved to the higher frequency (*δ* 7.7–7.9) in the (*E*)-isomer and conversely moved to the lower frequency (about *δ* 7.16) in the (*Z*)-isomer due to the weaker deshielding effect. In the HPLC analysis, the *cis-trans* isomers showed different polarities, and the peak of the (*E*)-isomer appeared later with a longer retention time due to its smaller polarity. In the UV analysis, the absorption peaks (λ_max_) of the (*Z*)-isomer showed a significant red shift in comparison with that of the (*E*)-isomer, and it was likely that the structure of the (*Z*)-isomer was spreading and had a stronger conjugation effect because of its two aromatic rings on different sides of the ethylene bridge. In the acidic aqueous solution, the (*E*)-isomer and (*Z*)-isomer can transform each other with increasing time.

It was found that FA had no inhibitory effect on RSV, but the A5 derived from FA significantly increased its anti-RSV effect with a TI value of 32, which was better than that of ribavirin (TI = 16). These derivatives did not work on EV71, and some of them had a weak anti-HSV-1 effect. FA introduced the aromatic ring at the α-position of carboxyl group, and modified by methylation, acylation, and esterification, *etc*., without an obvious rule between the structure and anti-RSV activity, but the specific A5 was found to have a selective anti-RSV effect. There was also no obvious difference in the antiviral activity of the cis-trans isomers. The A5 had no scavenging effect on DPPH· and ABTS+· because its phenolic hydroxyl group was acetylated, but A5 was easily decomposed to form A2 with better antioxidant effects. In addition, the A5 showed a proliferative effect on HUVECs. The A5 had a selective anti-RSV effect, and it can be used as a lead compound for further structural optimization by molecular docking and investigating the mechanism of inhibiting RSV and cardiovascular activity.

## Data Availability

The original contributions presented in the study are included in the article/Supplementary Material, further inquiries can be directed to the corresponding authors.
