# Supplementary figures and images for: Design, synthesis, antiviral activities of ferulic acid derivatives
Source: Front Pharmacol. 2023 Mar 3;14:1133655. doi: 10.3389/fphar.2023.1133655 (PMC10029727; doi:10.3389/fphar.2023.1133655)

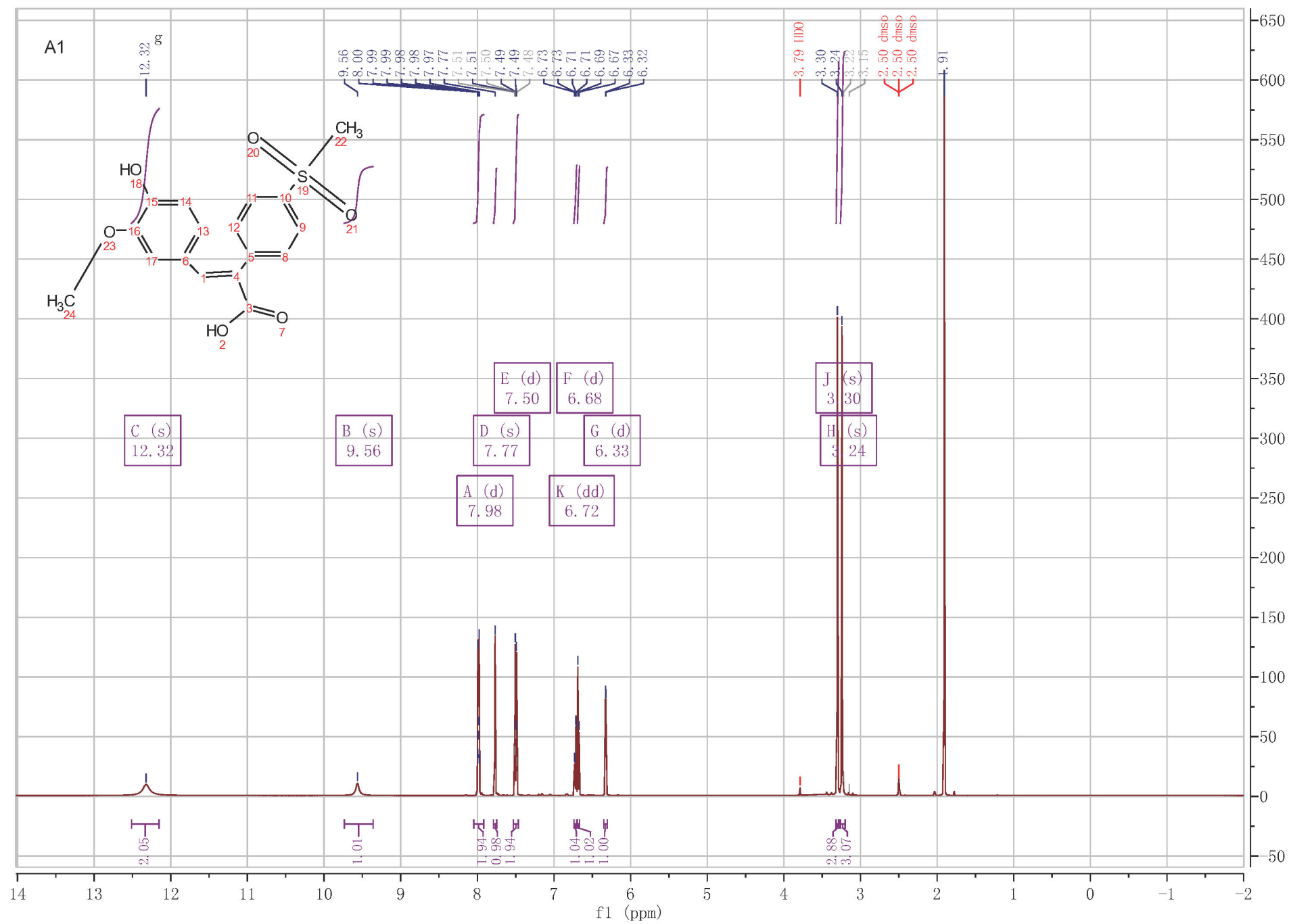

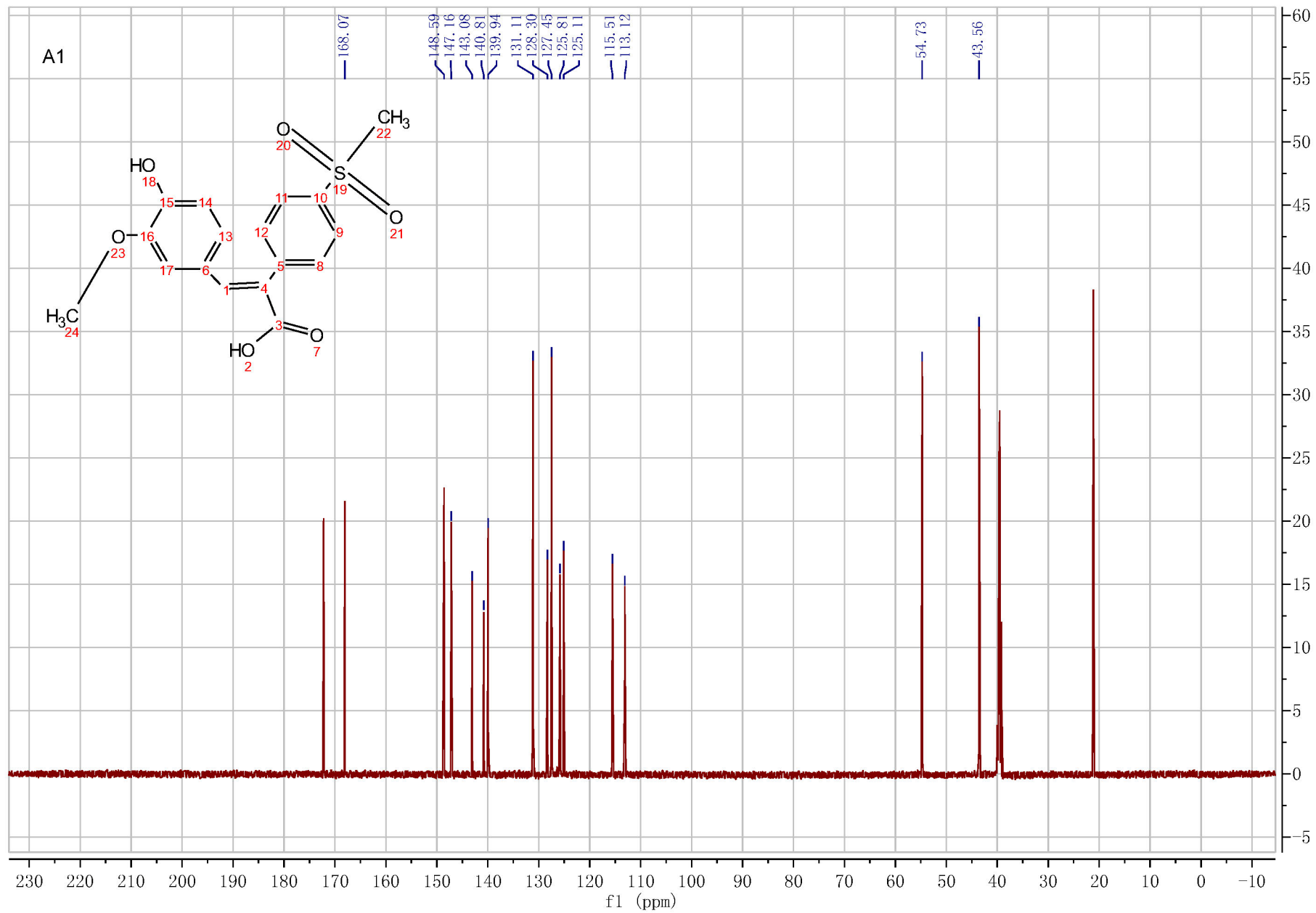

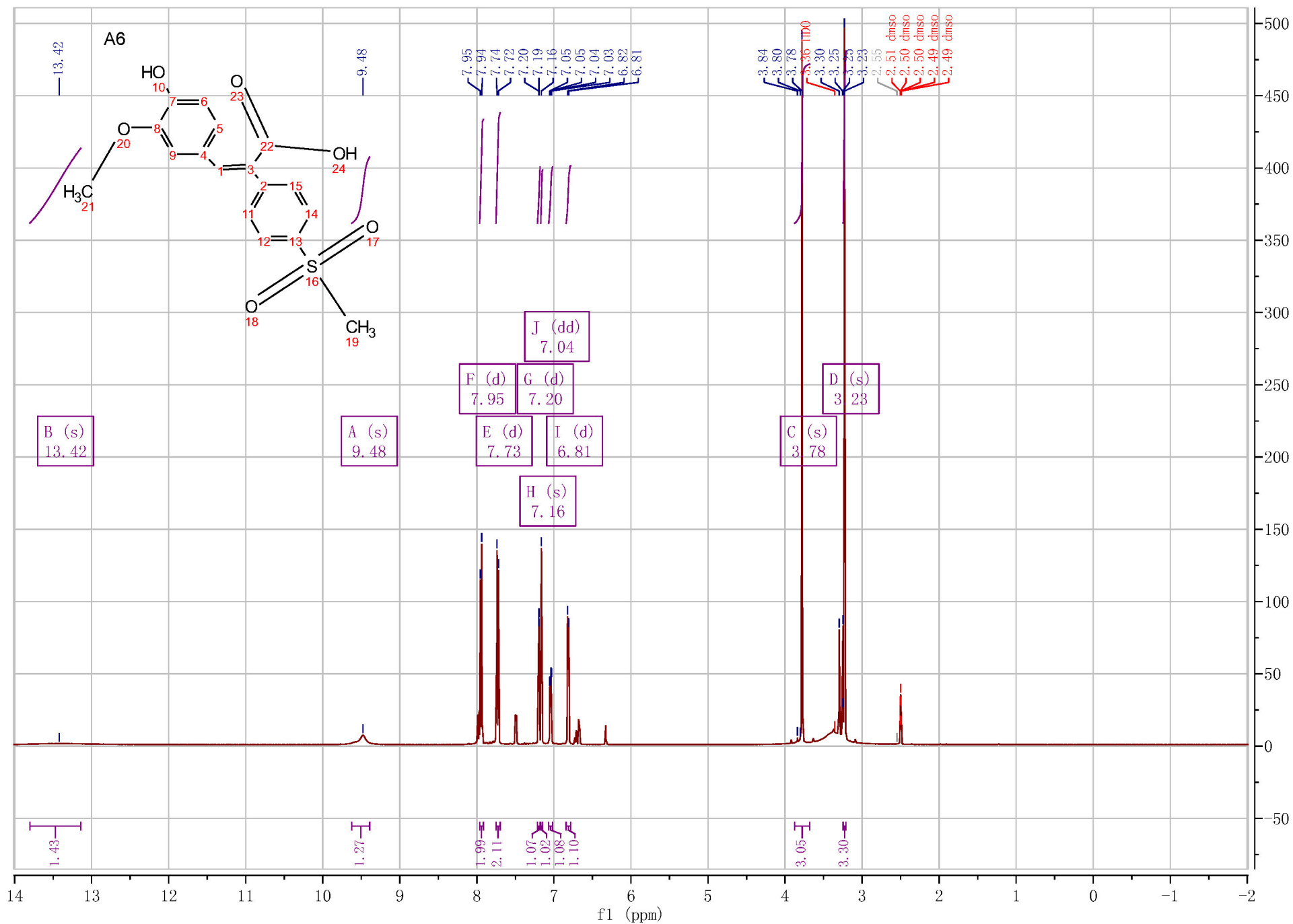

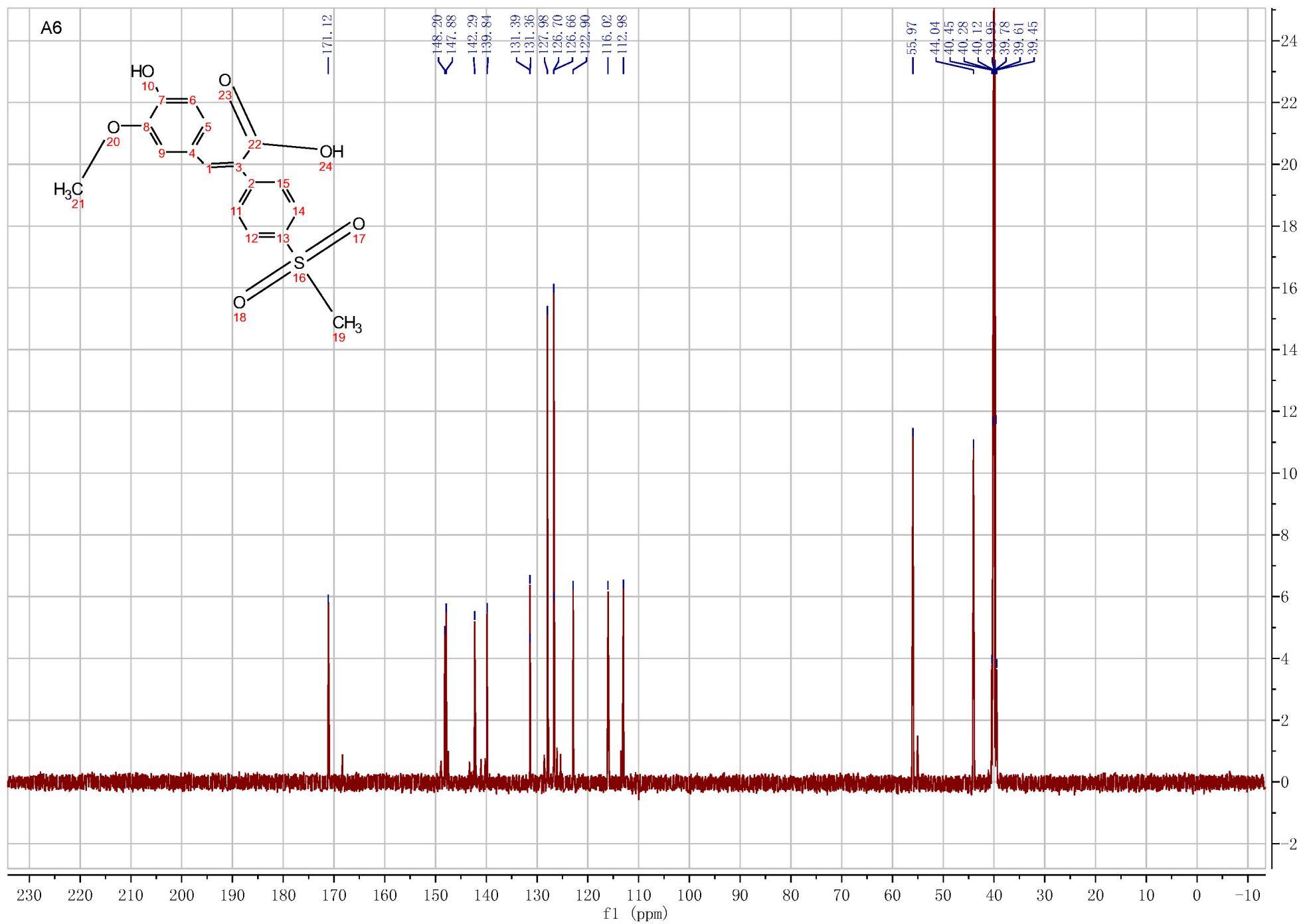

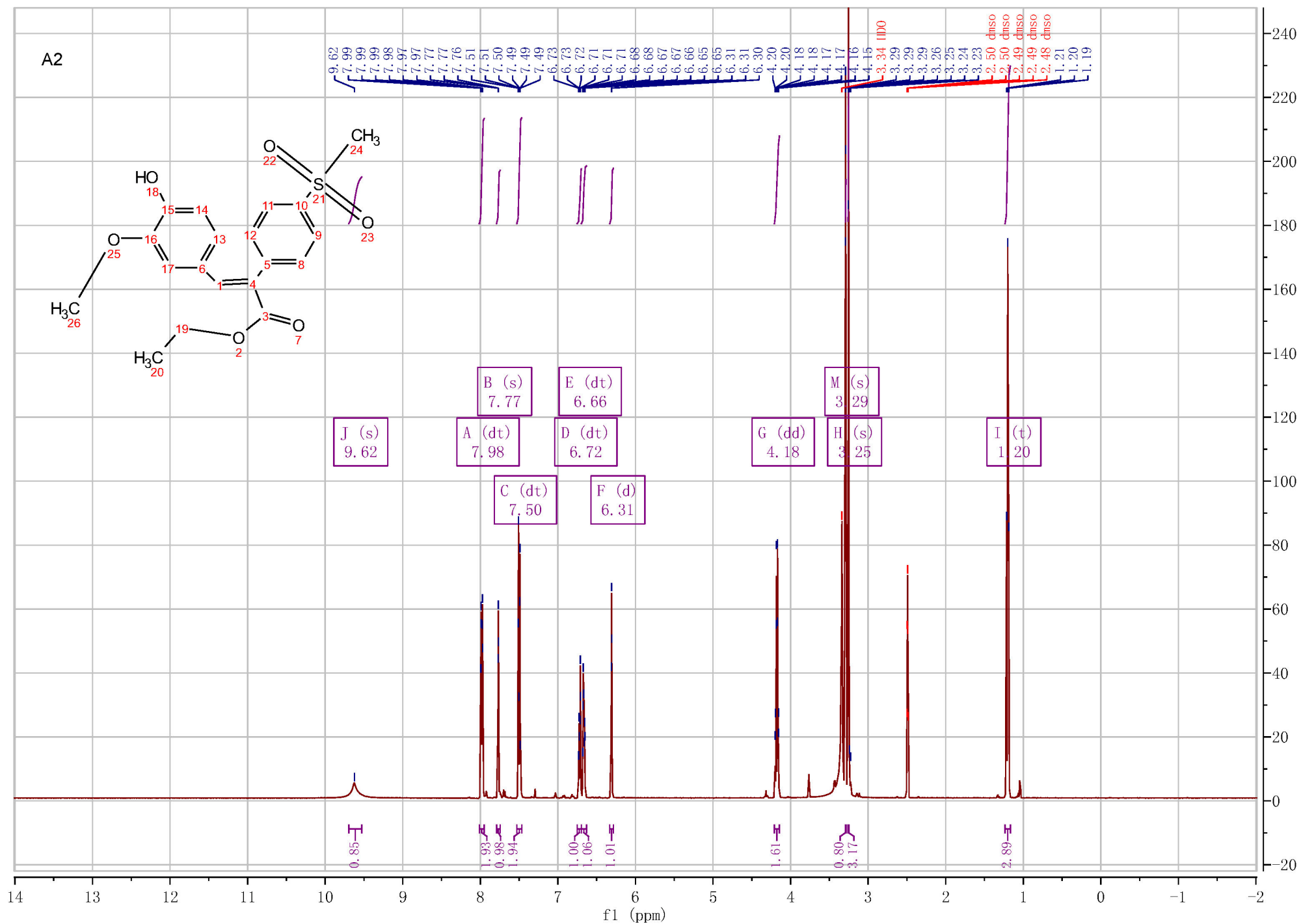

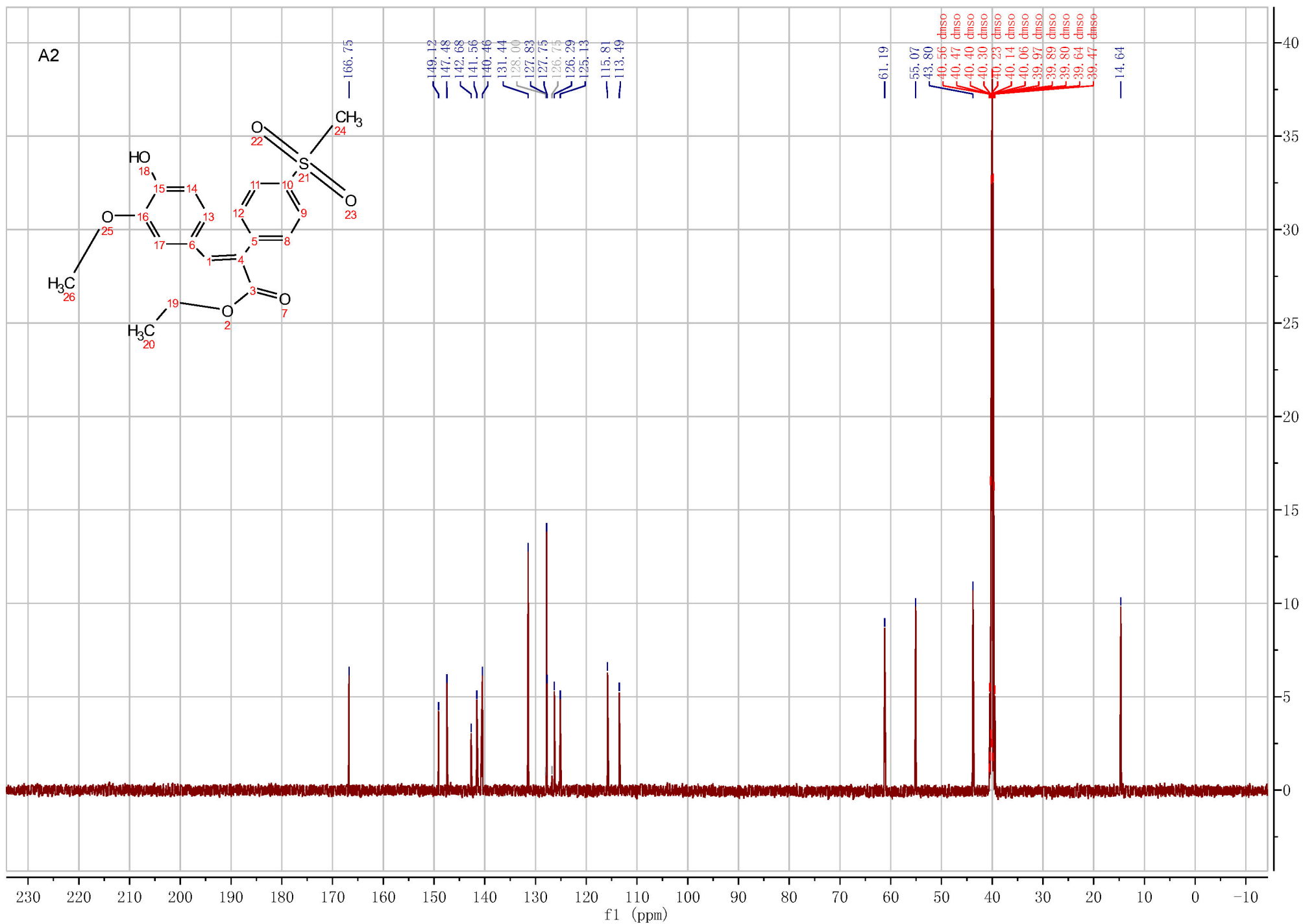

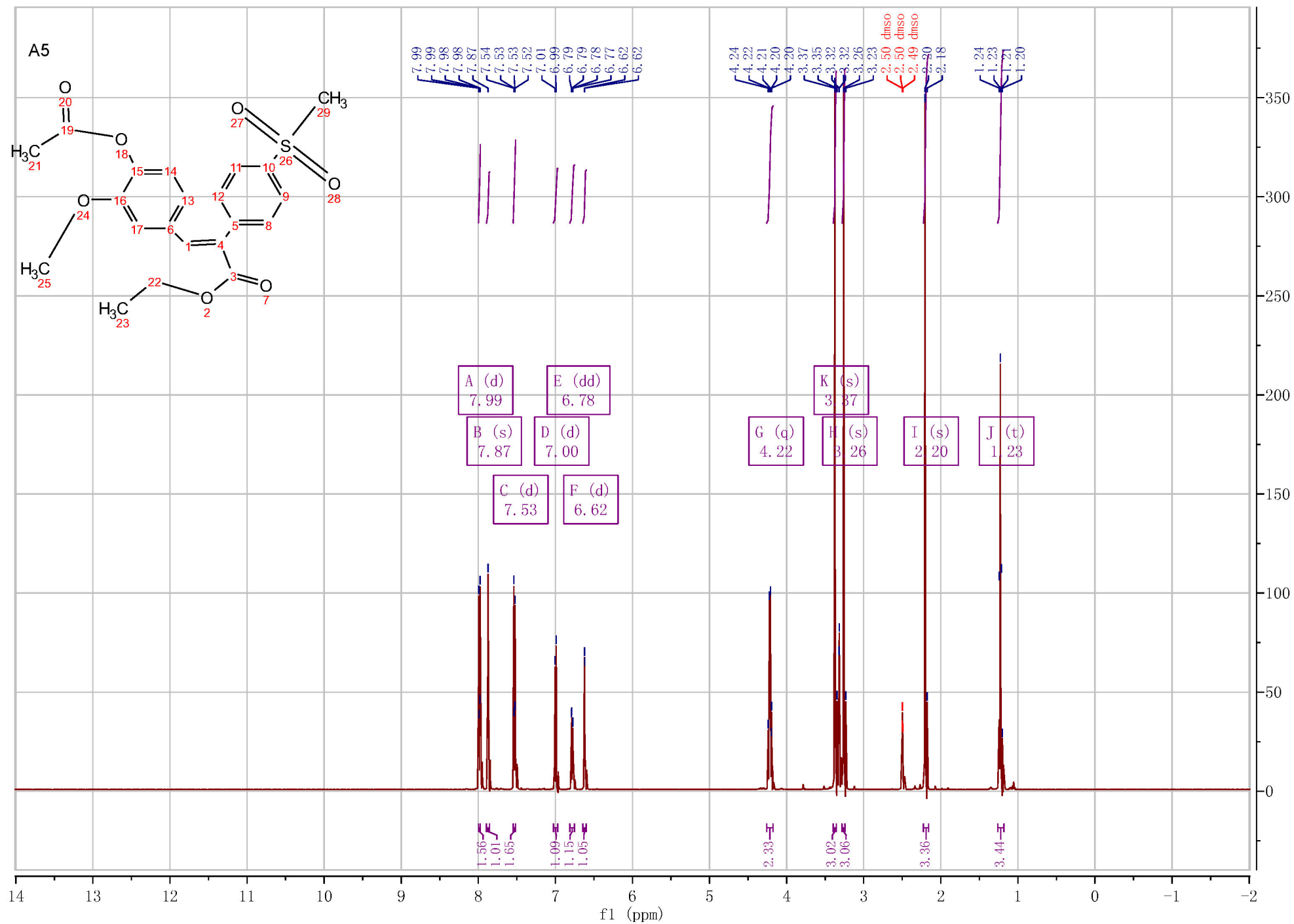

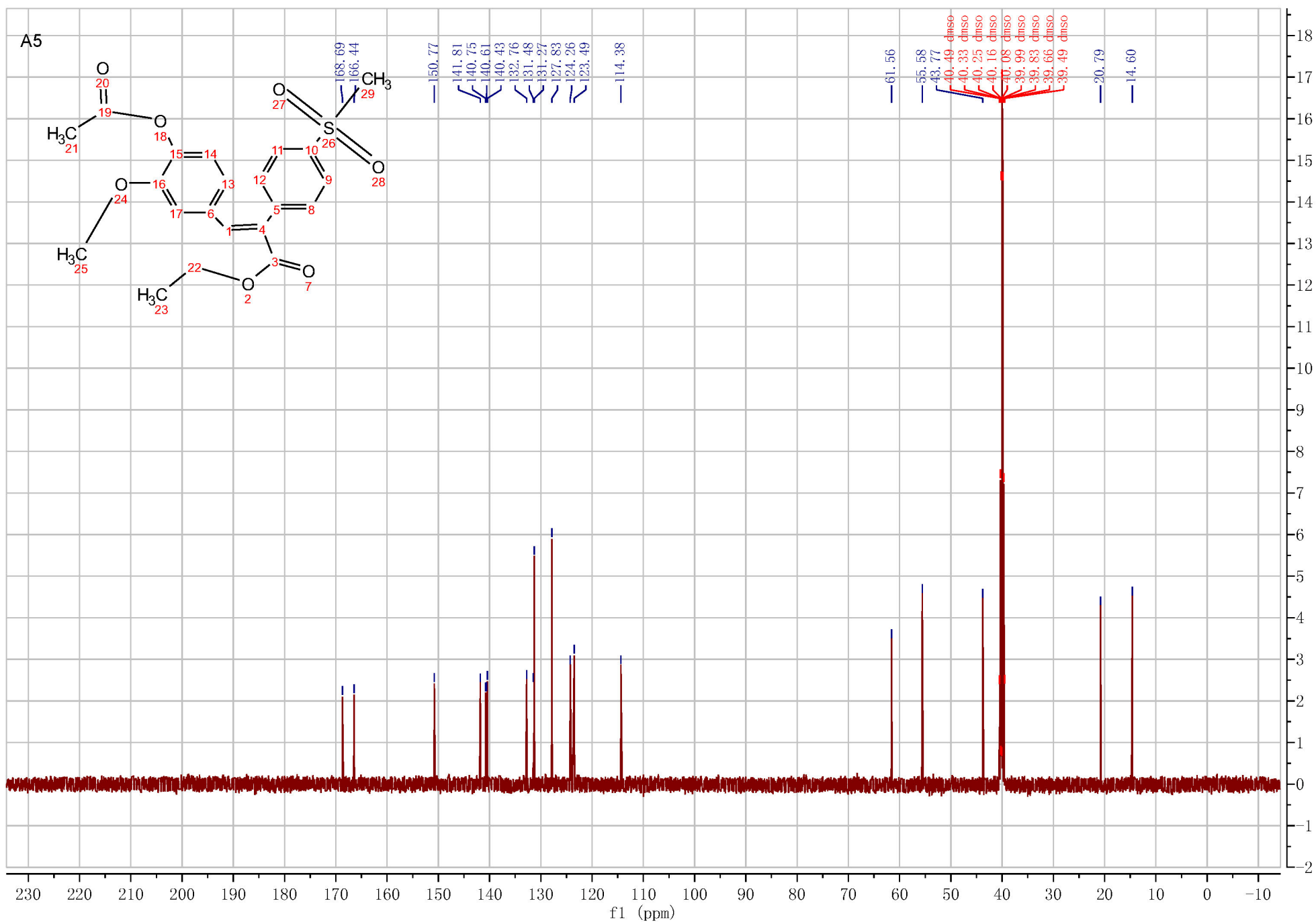

Supplement: Supplementary file 1 [file DataSheet1.PDF]
